# Estimating Environmental Transmission Risk From Host Movement Data

**DOI:** 10.1002/ece3.72571

**Published:** 2025-11-30

**Authors:** Himel Talukder, Ryan S. Miller, Raoul K. Boughton, Kurt C. Vercauteren, George Wittemyer, Kim M. Pepin, Anni Yang

**Affiliations:** ^1^ Department of Geography and Environmental Sustainability University of Oklahoma Norman Oklahoma USA; ^2^ United States Department of Agriculture Animal and Plant Health Inspection Service, Veterinary Services, Center for Epidemiology and Animal Health Fort Collins Colorado USA; ^3^ Mosaic Fertilizer LLC, Land and Resource Management Lithia Florida USA; ^4^ Archbold Biological Station Lake Placid Florida USA; ^5^ United States Department of Agriculture Animal and Plant Health Inspection Service, National Wildlife Research Center Fort Collins Colorado USA; ^6^ Department of Fish, Wildlife and Conservation Biology Colorado State University Fort Collins Colorado USA

**Keywords:** environmental transmission, host behavior, host movement, indirect contact, pathogen persistence

## Abstract

Environmentally mediated transmission is a critical pathway for disease spread, particularly for pathogens with environmental persistence. However, distinguishing which overlapping host movements result in transmission remains difficult, as most mobility models assume homogeneous exposure and overlook key spatial and behavioral factors. We extend previous models of indirect contact to account for variation in host movement behavior that could affect the probability of indirect transmission and examine these effects on transmission dynamics. We considered four models with different assumptions about the influence of movement behavior on indirect contact and transmission probability. The pathogen decay model includes all overlaps occurring within the pathogen's viability window. The high‐use area model restricts transmission to frequently visited locations, assuming greater pathogen deposition where hosts repeatedly congregate. The behavioral model filters contacts based on host behaviors relevant to deposition or acquisition. The integrated model incorporates both spatial and behavioral constraints. We applied these models to GPS telemetry data from wild pigs in Florida and simulated disease dynamics using SEIR models with environmental transmission for Influenza A and *Brucella suis* , representing pathogens with short and long environmental persistence, respectively. Compared to pathogen decay model, total contact numbers decreased by 0.3% in high‐use model, 70.3% in behavioral model, and 70.4% in integrated model under the short‐lived condition. Edge density declined from 0.60 to 0.50, transitivity from 0.81 to 0.77, and assortativity from 0.43 to 0.17. *R*_*0*_ declined from 2.42 ± 0.47 to 2.06 ± 0.39 (high‐use), 1.56 ± 0.42 (behavioral), and 1.35 ± 0.40 (integrated), with a lag of approximately 21 days in peak incidence. The long‐lived scenarios, were quantitively similar to those of short‐lived scenarios. These results underscore that assumptions about how indirect contact from movement data maps to pathogen transmission significantly influence network configuration and epidemic projections. Our findings highlight the importance of mechanistically defining indirect transmission in disease modeling.

## Graphical abstract

We present a comparative modeling framework that integrates host movement, pathogen persistence, and behavior to quantify environmentally mediated transmission. Using wild pig GPS data and SEIR simulations, we show that contact definitions significantly alter transmission dynamics, highlighting the importance of mechanistically defining indirect contacts in ecological disease models.

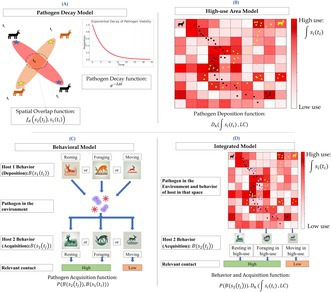

## 1 Introduction

Pathogens and parasites can spread through multiple routes of exposure, including contact among hosts (Craft 2015; Parratt et al. 2016; Gwenzi et al. 2022). Direct contact transmission occurs when hosts engage in physical contact (White et al. 2017; VanderWaal and Ezenwa 2016). Pathogen transmission can also occur indirectly through vectors and fomites, mediated through environmental factors or mechanical vectors (Fofana and Hurford 2017). Many pathogens, such as *Mycobacterium bovis* , *Brucella* spp., and *influenza A viruses*, can survive in the environment, and be transmitted via host contact with contaminated surfaces, soil, water, or other environmental media (Pandey et al. 2024; Suh et al. 2024). This process, referred to as environmentally mediated transmission, can be a primary transmission pathway for those disease systems and plays an important role in facilitating pathogen spread among social groups of hosts. Despite its recognized importance in disease ecology, environmentally mediated transmission remains less well‐characterized than direct transmission and vector‐borne transmission, largely due to the challenges of identifying environmentally mediated contacts (Collier et al. 2022).

Historically, contact networks have been quantified with a variety of direct and indirect observational and technological tools (Arthur et al. 2017). Camera trap data are useful for determining species composition, group size, and coarse contact patterns (Herraiz, Ferrer‐Ferrando, et al. 2024; Smith, Snyder, and Owen 2020; Smith, Suraci, et al. 2020; Kukielka et al. 2013). However, they are limited in terms of spatial and temporal continuity, and they might not capture the individual‐level movements required to model environmental exposure to pathogens (Triguero‐Ocaña et al. 2021; Egan et al. 2023). Proximity loggers can track individuals' physical closeness at a continuous temporal scale (Yang, Proffitt, et al. 2021; Yang, Schlichting, et al. 2021; Pepin et al. 2021); however, they are limited to capturing environmentally mediated contacts only via known point sources when the stationary loggers were deployed at pre‐defined locations (Kelt et al. 2019).

Wildlife telemetry offers rich spatiotemporal movement data and has been increasingly used to study individual–environment interactions (Noonan et al. 2021). The ubiquity of movement data in wildlife studies provides the opportunity to capture spatially explicit direct and indirect contacts. Specifically, direct contact is often defined as colocation at the same time, while environmentally mediated contact is defined as colocation at segregated times (Kenward et al. 1998; Long et al. 2022). Nevertheless, not all instances of colocation at segregated times are epidemiologically relevant (Dougherty et al. 2018). For instance, a host simply passing through a location previously visited by another host may rarely result in pathogen transmission. The success of environmentally mediated transmission can be affected by various factors, including pathogen persistence in the environment, host immune response, level of environmental contamination, and host acquisition behavior (Rees et al. 2021).

One of the principal challenges in defining environmentally mediated contact is distinguishing between incidental spatial overlap and contacts that pose a meaningful risk for pathogen transmission (Manlove et al. 2022). Thus, identifying relevant environmentally mediated contact requires the consideration of the factors that trigger and affect the transmission. For example, in environmentally persistent diseases like brucellosis, an infected host may deposit pathogens in a shared environment where they remain viable for days to weeks, allowing susceptible individuals to acquire the pathogen upon subsequent visitation (Salman and Steneroden 2022). However, if a susceptible host arrives at the site after the pathogen has decayed beyond infectious viability, the contact is no longer relevant (Daversa et al. 2017; Richardson and Gorochowski 2015). Additionally, behavioral heterogeneity among hosts can add further complexity (Egan et al. 2023; Clontz et al. 2021). Certain behaviors, such as foraging, resting, drinking, or wallowing, may increase the likelihood of pathogen deposition and acquisition, while transient movement through contaminated areas may not provide sufficient exposure for transmission (Dougherty et al. 2022; Binning et al. 2017). Existing research (Yang et al. 2023; Wilber et al. 2022), tends to presume that all spatiotemporal overlaps correspond to equivalent transmission risk. This is a simplification of environmentally mediated transmission that overlooks behavioral context and pathogen persistence. Our framework fills this gap by adding these dimensions and adjusting their effect on disease spread.

Previous research on indirect contact that relies on spatiotemporal overlap frequently makes the assumption that every instance of non‐simultaneous co‐location contributes equally to the risk of transmission (Dougherty et al. 2018; Yang et al. 2023). However, this method ignores epidemiological factors that influence pathogen deposition and acquisition likelihood, such as host movement behaviors and pathogen survival duration. These oversimplified overlap models may therefore greatly overestimate the risk of indirect transmission, particularly for pathogens that need particular behavioral or environmental factors to spread successfully. To address these limitations, an integrative approach is required to systematically quantify relevant environmentally mediated contacts by incorporating host movement data, pathogen survival dynamics, and behavioral ecology. This modeling approach is a continuation of earlier foundational work that utilized movement data to construct spatio‐temporally varied exposure networks (Wilber et al. 2022; Yang et al. 2023), which has recently been compared against baseline proximity‐based measures of risk in empirical disease networks (Herraiz, Triguero‐Ocaña, et al. 2024). They have illustrated how contact modeling frameworks can provide us with valuable information on transmission and force of infection based on pathogen decay and host movement data.

However, previous uses were limited in scope. For example, they often primarily focused on pathogen decay without explicitly parameterizing deposition or acquisition processes in empirical systems, or they lacked a systematic investigation of how varying mechanistic assumptions influence network structure or epidemic dynamics (Wilber et al. 2022; Yang et al. 2023; Herraiz, Triguero‐Ocaña, et al. 2024). In the present study, we expand on this framework to explore how mechanistic filters, such as behavioral state, pathogen decay dynamics, high‐use area assignment, and spatial thresholds, interact to build exposure networks and epidemic impacts. Because of the critical role of pathogen ecology in translating indirect contact, this modular comparison is best suited to environmentally persistent pathogens, where contaminating and acquiring assumptions largely predominate transmission dynamics. The objective of this study is to develop a methodological framework to test hypotheses about the mechanisms that drive relevant indirect contact based on host movement data under different transmission scenarios. Specifically, we integrate host behaviors, pathogen survival times, and environmental contaminations to estimate dyadic exposure levels related to disease transmission risk. This framework will allow for more precise characterization of environmentally mediated contact events distinguishing those that are likely to facilitate pathogen (e.g., virus, bacteria, and fungi) from those that are not. Contrasting outputs from these different models allow identification of conserved or sensitive transmission properties subject to these different processes. By integrating environmental dynamics with classical compartmental transitions, this framework not only enables retrospective consideration of environmental transmission but also comparative evaluation across host‐pathogen systems with contrasting ecological features.

## 2 Methodology

### 2.1 Modeling Framework

**FIGURE 1:**
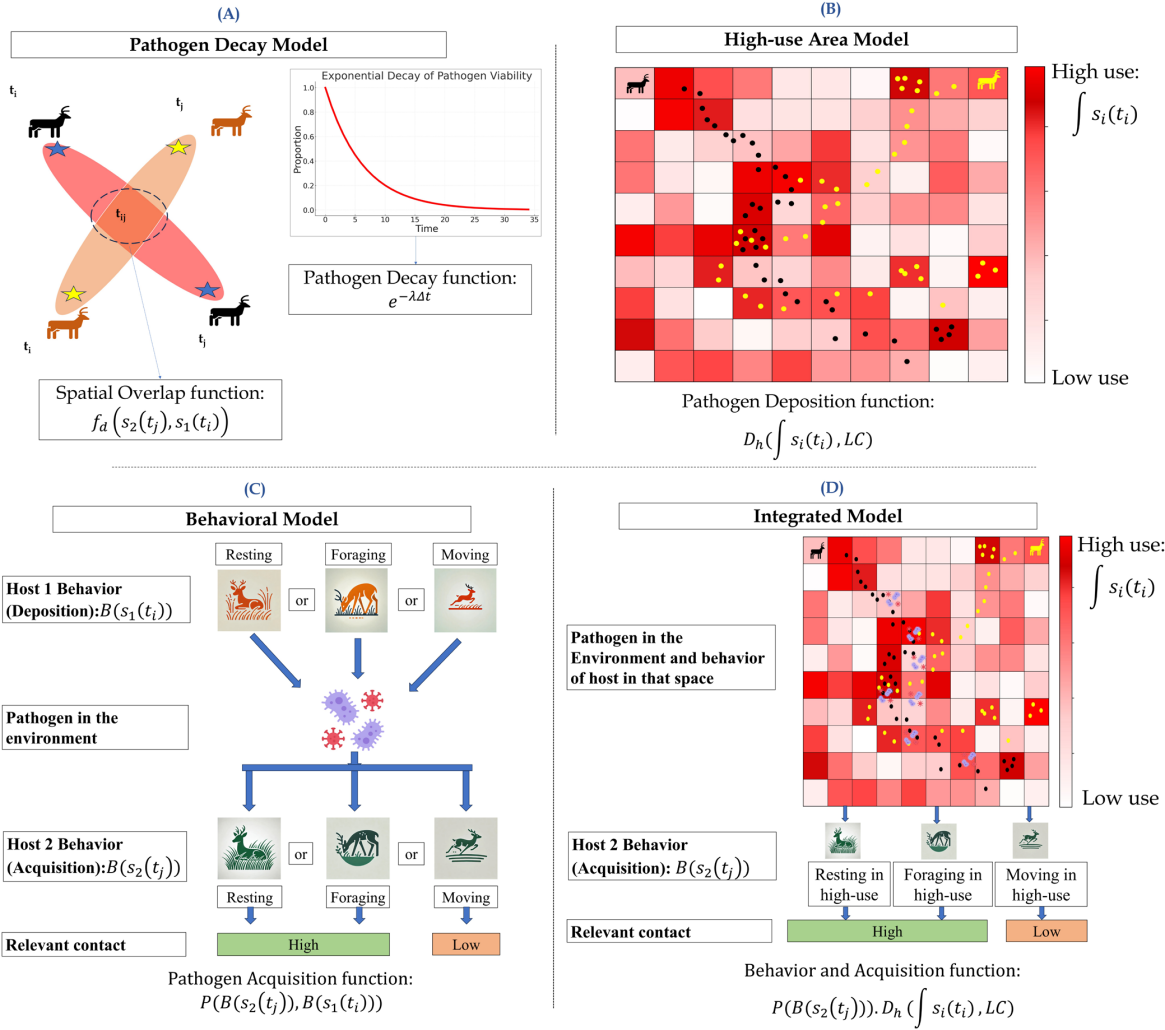
Methodological framework for capturing relevant environmentally mediated contact; (A) Quantifying potential exposure levels relating pathogen decay to the temporal lag in spatial overlap, (B) Estimating pathogen deposition levels based on time spent by infected individuals in a given location, (C) Adjusting exposure estimates by incorporating behavioral modes that influence pathogen deposition and/or acquisition and (D) integrating deposition‐relevant space use and acquisition‐relevant behaviors into a spatial representation of transmission risk.

Building on the framework proposed by Wilber et al. (2022) and extended by Yang et al. (2023), we developed four distinct approaches to leveraging movement data to inform indirect transmission probability, that is, pathogen decay model, high‐use model, behavioral‐state model, and integrated model (Figure 1).

According to Yang et al. (2023), the movement trajectory for the *k*th host in the disease system is denoted as *s*_*k*_(*t*), where *s* is a two‐dimensional vector of the host's spatial coordinates, and *t* represents time. The instantaneous weight of environmentally mediated contact $\left(w_{i,j}^{1\to 2}\right)$ between Host 1 at time *t*_*i*_ and Host 2 at time *t*_*j*_ is determined by the time difference (Δ*t*) between *t*_*i*_ and *t*_*j*_ and the distance (*d*) and only *j* < *i* is considered, that is, only past exposures are considered. This creates the constraint that indirect contact can occur only if there is a time lag between deposition and acquisition. Direct contact (i.e., *j* = *i*) is excluded by definition in this model. The equation is given by:

(1) $$w_{i,j}^{1\to 2}=\underset{\text{Temporal encounter}\;}{\underbrace{f_{\delta }\left(\mathrm{\Delta t}=t_{j}-t_{i}\right)}}.\underset{\text{Spatial encounter}}{\underbrace{f_{d}\left(s_{2}\left(t_{j}\right),s_{1}\left(t_{i}\right)\right)}};t_{i}<t_{j}$$

where $f_{\delta }$ is a temporal encounter function and $f_{d}\left(s_{2}\left(t_{j}\right),s_{1}\left(t_{i}\right)\right)$ determines spatial distance threshold for contact, and that determines the temporal gap of the contact. The functions (*f*_*d*_ and *f*_*δ*_) that relate time and distance between indirect contacts to risk of transmission can be defined by the user to reflect the nuances of the host‐pathogen system. In Equation (1), we used binary step functions to express that there is a contact when the trajectories are within a specific time and distance, and not otherwise. In the subsequent models, this framework is modified or extended by incorporating terms for pathogen deposition, behavioral acquisition, or their contact. A contact kernel $K^{1\to 2}$ experienced by Host 2 from Host 1 consists of the instantaneous weight of contact across all timesteps:

(2) $$K^{1\to 2}=\left[\begin{array}{ccccc} 0 & w_{1,2}^{1\to 2} & w_{1,3}^{1\to 3} & \cdots & w_{1,n}^{1\to n}\\ 0 & 0 & w_{2,3}^{1\to 2} & \cdots & w_{2,n}^{1\to 2}\\ 0 & 0 & 0 & \cdots & \vdots \\ \vdots & \vdots & \vdots & \ddots & w_{n-1,n}^{1\to 2}\\ 0 & 0 & 0 & \cdots & 0 \end{array}\right]$$

#### 2.1.1 Pathogen Decay Model

For highly transmissible pathogens that are transmitted via the environment (e.g., Influenza A virus; Leung 2021), the inhalation or contact with virus‐laden aerosols or respiratory droplets might trigger infection, regardless of host acquisition or deposition behaviors (Wang et al. 2021). In such scenarios, the transmission rate of the disease systems is primarily impacted by the pathogen decay rate in the environment, as pathogens may remain viable only for specific periods based on environmental conditions like temperature and humidity (Wißmann et al. 2021). Several studies have suggested that the likelihood for many pathogens to remain infectious in the environment declines exponentially over time (Laggan et al. 2023), though we note exceptions to this are known (e.g., CWD). To estimate the environmentally mediated contact weights based on the potential transmission risk influenced by the natural loss of pathogen viability, we parameterize the spatial encounter function as an exponential decay function (Figure 1):

(3) $$w_{i,j}^{1\to 2}=\underset{\text{Spatial encounter}}{\underbrace{f_{d}\left(s_{2}\left(t_{j}\right),s_{1}\left(t_{i}\right)\right)}}.\underset{\text{Pathogen Decay}}{\underbrace{e^{-\lambda \Delta t}}}$$

where λ is a parameter that defines the rate at which the pathogen decays in the environment.

#### 2.1.2 High‐Use Area Model

For some environmentally mediated disease systems, such as those involving *Leptospira* spp., which primarily rely on exposure to contaminated environments, it is essential to identify the high‐risk areas for potential contamination (Bradley and Lockaby 2023). Additionally, in dry climates with limited water resources, animals may congregate around small water sources, such as livestock water supplies, leading to pathogen accumulation and increased disease transmission risk. Based on host movement patterns, it has been hypothesized that areas with higher visitation frequencies and longer durations of stay are more likely to be contaminated by hosts (Esposito et al. 2023). These areas increase transmission risk, as repeated contacts with the contaminated environment facilitate pathogen transfer between individuals (Plowright et al. 2017; Pepin et al. 2021). Building on the pathogen decay model, we incorporate an additional dimension by considering potential risk areas for pathogen deposition. Specifically, we define high‐use areas, which are locations that animals visit frequently as potential contamination hotspots. Additionally, in some scenarios, certain point resources or specific habitats may be known where the shedding host is more likely to shed pathogens, such as the water ponds in avian influenza transmission. To better capture environmentally mediated contact events relevant to transmission in such disease systems, we adjust the environmentally mediated contact weights based on potential pathogen deposition. The equation for this high‐use area model is defined as:

(4) $$w_{i,j}^{1\to 2}=\underset{\text{Spatial encounter}}{\underbrace{f_{d}\left(s_{2}\left(t_{j}\right),s_{1}\left(t_{i}\right)\right)}}.\underset{\text{Pathogen Decay}}{\underbrace{e^{-\lambda \Delta t}}}.\underset{\text{Pathogen Deposition}}{\underbrace{D_{h}\left(\int s_{i}\left(t_{i}\right),\mathrm{LC}\right)}}$$

where $D_{h}\left(\int s_{i}\left(t_{i}\right),\mathrm{LC}\right)$ is a function of pathogen deposition based on the cumulative visitations of the host on the landscape ($\int s_{i}\left(t_{i}\right)$), that is, the high‐use areas. Landscape features (LC) are set up to act as a feature‐presence‐associated constant, enabling the introduction of habitat‐based deposition constraints where existing knowledge warrants those associations. Habitat‐based variation in pathogen persistence or host behavior would add a substantial amount of uncertainty to our inferences. Here, LC functions as a spatial mask, allowing contacts to be evaluated only within a specified land‐cover type.

#### 2.1.3 Behavioral Model

The Behavioral Model highlights how specific behaviors impact disease transmission. Certain behaviors of Host 2 (the recipient), such as resting and foraging, can increase the chance of pathogen acquisition from the environment, making those indirect contacts relevant (Dougherty et al. 2022). In contrast, Host 2 with moving or walking behavior is considered irrelevant for transmission, as transient environmental interactions do not provide sufficient exposure. This distinction is particularly important in disease systems where transmission depends on behaviors like grazing or nesting (e.g., anthrax) (Binning et al. 2017; Yang, Proffitt, et al. 2021; Yang, Schlichting, et al. 2021). Additionally, some behaviors might contribute to more pathogen shedding in the environment than others. For example, in some fecal–oral transmission systems like chronic wasting disease, the foraging or licking behaviors by the shedders (Host 1) can cause environmental contamination (Otero et al. 2021). Host movement data can be used to probabilistically distinguish these behaviors, allowing for the better identification of relevant environmentally mediated contact. The equation for this model is:

(5) $$w_{i,j}^{1\to 2}=\underset{\text{Spatial encounter}\;}{\underbrace{f_{d}\left(s_{2}\left(t_{j}\right),s_{1}\left(t_{i}\right)\right)}}.\underset{\text{Pathogen Decay}}{\underbrace{e^{-\lambda \Delta t}}}.\underset{\text{Pathogen Acquisition and Deposition}}{\underbrace{P\left(B\left(s_{2}\left(t_{j}\right)\right),B\left(s_{1}\left(t_{i}\right)\right)\right)}}$$

where $P\left(\left(B\left(s_{2}\left(t_{j}\right)\right)\right),B\left(s_{1}\left(t_{i}\right)\right)\right)$ is a probability function that defines the likelihood of transmission based on different acquisition and deposition behaviors, with a primary focus on the behavior of Host 2 (the acquiring individual) at the time of environmental contact. $B\left(s_{2}\left(t_{n}\right)\right)$ is a function that estimates the behavioral states of Host 1 and/or 2 based on its trajectory at that time. For example, *P* can be a piecewise function: when Host 1 and 2 are estimated as moving, the probability can be low, as this behavior is not related to disease transmission. Conversely, if Host 1 and 2 are estimated to be foraging or resting, we can assess a high probability, reflecting behaviors relevant to pathogen acquisition (Egan et al. 2023).

#### 2.1.4 Integrated Model

There are also some fecal–oral disease systems such as bovine brucellosis; the major shedding route—contamination via body fluid and fetus during abortion—is hard to predict or capture by just using host movement data (Khurana et al. 2021). However, it is critical to consider pathogen deposition and acquisition to identify relevant environmentally mediated contacts in such disease systems. Thus, we propose to integrate the high‐use model and behavioral model to estimate the potential areas of high contamination and estimate the likelihood of hosts acquiring pathogens from the environment through specific behaviors, enhancing the refinement of relevant environmentally mediated contacts. A contact is retained only if it satisfies both spatial and behavioral conditions—occurring in a high‐use area and involving behavior combinations that elevate transmission risk. Compared to the earlier models reliant on single alterations to the base contact definition, the integrated model includes spatial and behavioral constraints, extending the function initially proposed in Equation (1). Our framework builds on prior work (Wilber et al. 2022; Yang et al. 2023), by combining behavior filtering and high‐use environmental weighting into a modular structure, which allows these components to be toggled and compared independently to facilitate flexible, scenario‐based estimation of environmentally mediated transmission under different scenarios. The equation for this model is:

(6) $$w_{i,j}^{1\to 2}=\underset{\text{Spatial encounter}}{\underbrace{f_{d}\left(s_{2}\left(t_{j}\right),s_{1}\left(t_{i}\right)\right)}}.\underset{\text{Pathogen Decay}}{\underbrace{e^{-\mathrm{\lambda \Delta t}}}}.\underset{\text{Pathogen Deposition}}{\underbrace{D_{h}\left(\int s_{i}\left(t_{i}\right),\mathrm{LC}\right)}}.\underset{\text{Pathogen Acquisition}}{\underbrace{P\left(B\left(s_{2}\left(t_{i}\right)\right)\right)}}$$

The equation is interpreted in a sequential process that governs environmentally mediated transmission. The term *D*_*h*_ indicates the deposition of pathogens by Host 1 upon shedding, and the behavioral probability function *P*(*B*) controls the chance of Host 2 acquisition upon subsequent environmental contact. Although the behavioral filter can theoretically include both hosts, our implementation targets conditioning to the behavior of the acquiring host because deposition is automatic when Host 1 visits the site, yet acquisition depends on the behavioral status of Host 2 upon contact with the contaminated environment.

### 2.2 Case Study

#### 2.2.1 Data Description and Study Area

To demonstrate the applicability of our methodological framework, we applied it to determine transmission relevant environmentally mediated contacts in the wild pig ( *Sus scrofa* ) population in Florida. Wild pigs exhibit a complex social structure, typically forming matriarchal groups consisting of adult females and their offspring, while adult males are often solitary or form small bachelor groups (Gabor et al. 1999; Kaminski et al. 2005; Clontz et al. 2023). This social behavior influences their movement patterns and contact dynamics, making them an ideal species for studying environmentally mediated disease transmission (Yang et al. 2023; Podgórski et al. 2014). We captured 17 adult wild pigs (10 female, 7 male) from different social groups on the Archbold Biological Station—Buck Island Ranch (ABIR) in Florida, USA and deployed GPS collars (Catlog GPS device and Lotek LMRT3 VHF Collars, Lotek, WA, US) to collect their movement trajectories from December 13, 2019 to September 13, 2020, with 16 days of missing information in between, thus having 259 days of data. Despite the fact that the subjects were not collared on the same day and tracking sessions differed slightly, we restricted our analysis to the overlap duration December 13, 2019 to September 13, 2020 when all 17 pigs were being tracked simultaneously so that we could have uniform temporal coverage for pairwise contact analysis. ABIR is a 42.3 km^2^ commercial beef cow‐calf operation managed at commercial production levels and maintains an average standing inventory of ~3000 head of cattle. The details of the study site can be found at (Yang et al. 2023). The collars were programmed to record GPS fixes every 10 min with locational errors of 6–10 m on average. To test our models, we have also simulated pathogen transmission of swine influenza virus for short‐lived (7 days) and brucellosis for long‐lived (30 days).

#### 2.2.2 Model Parameterization

For the parameterization of the pathogen decay model, we define the spatial encounter function, $f_{d}\left(s_{2}\left(t_{j}\right),s_{1}\left(t_{i}\right)\right)$, as a piecewise function with a 10‐m distance threshold (Yang et al. 2023). Specifically, we define $f_{d}$ (*d*) = 1 if *d* ≤ 10 m, and 0 otherwise; reflecting that contacts only occur within 10 m, a threshold chosen to match the higher limit of GPS error range (6–10 m). This accounts for location inaccuracies, ensuring identified co‐locations are realistic and while accounting for locational uncertainty and reducing the likelihood of both false‐positive and false‐negative contact assignments. To consider the reduction in pathogen viability over time, we used two decay rates of *λ*_1_ = 0.7 per day and *λ*_2_ = 0.16 per day. These rates represent pathogens with different environmental persistence, allowing us to demonstrate and compare how environmentally mediated contact kernels and distributions differ across disease systems. The decay rates assume an exponential decline in pathogen viability, with *λ*_1_ = 0.7 resulting in nearly complete decay of pathogens within 7 days, while *λ*_2_ = 0.16 reflecting a slower decay, leading to negligible pathogen presence after 30 days (Figure S1).

In the high‐use model, we defined *D*_*h*_ (deposition of pathogens) as high‐use areas, which were parameterized based on host visitation rates. The study area was divided into a 30 × 30‐m grids to match the spatial resolution of standard land cover products, such as the National Land Cover Database (NLCD) for facilitation of future fusing with environmental covariates. We used GPS positional uncertainty (~10 m) as a guide for deriving the minimum practical grid size, but edge effects are still problematic at any resolution. However, land cover was not explicitly incorporated in this case study to maintain focus on behavioral dynamics and due to limited habitat‐specific shedding data; following prior work (Yang, Proffitt, et al. 2021; Yang, Schlichting, et al. 2021; Wilber et al. 2022), incorporating land cover as a spatial weight in future models could enhance predictive accuracy by accounting for habitat‐driven variation in host movement and environmental persistence. Land cover filtering in systems where pathogen deposition or persistence is understood to depend on particular landscape features is straightforward for the model to accommodate, but for this case study, we focused on only for clear interpretation of these effects on indirect contact. A visit is defined as an occurrence of an animal spending time in a specific location with at least one GPS point recorded, where successive visits to the same spatial unit are separated by a temporal gap of at least 10 min. Visitation rates were defined as the number of times hosts visited a given spatial zone (each grid cell), normalized by the total observation period. High‐use areas were defined as locations within the 75th percentile of visitation rates, representing zones with elevated host activity. These areas are assumed to have higher levels of environmental contamination due to frequent deposition and acquisition events, making them critical hotspots for pathogen transmission. Rather than weighting the deposition process towards higher or lower rates, these high‐use zones act as spatial filters; a contact is only kept if it takes place inside one of these zones. We also tested the 50 and 90 percentiles to define the high‐use area and how these thresholds influence the identification of relevant environmentally mediated contacts (see Figures S5 and S6).

In the behavioral model, the behavioral states based on host movement were classified using a Hidden Markov Model (HMM), which defines stages based on step length and turning angle derived from GPS movement data (Rabiner and Juang 1986; Awad and Khanna 2015). We parameterized the Hidden Markov Model (HMM) using a Gaussian distribution with three behavioral states, incorporating step length and turning angle as observational features derived from normalized spatial and temporal GPS data. Each model was trained individually for each animal, utilizing iterative optimization with up to 200 iterations to ensure convergence by checking changes in log‐likelihood. Sex of the animal was included as a covariate due to differences in sex‐specific behavior and movement patterns in wild pigs (Clontz et al. 2021), enabling the model to take into consideration variations in male and female step length, turning angle, and state transition probabilities. The Viterbi algorithm was employed to decode the most probable state sequence for each trajectory and then the states were assigned to each of the GPS points, facilitating biologically meaningful classification of movement behaviors (Lou 1995; Clontz et al. 2021). A schematic of the Hidden Markov Model structure, including transition probabilities, step length & turning angle distributions, is provided in Figure S7. We have used *hmmlearn* package in Python library for these analyses. For the probability function $P\left(B\left(s_{2}\left(t_{j}\right)\right)\right)$, which defines the likelihood of transmission based on different acquisition and deposition behaviors, we used a simplified parameterization where the probability of transmission is set to 1 for foraging and resting states and 0 for the moving state. We kept foraging and resting states as separate classes to demonstrate the flexibility of our approach for future applications using higher‐resolution behavioral data, even though they were given equal transmission probabilities in this implementation. Finally, the integrated model combined the parameterizations of high‐use and behavioral models. While continuous filters can be included in the framework, in this case study, the filters are binary and serve as filters to find pertinent contacts. This simplification makes it possible to interpret the relative influence on contact more clearly.

#### 2.2.3 Disease Transmission Model

To illustrate the implications of our contact models for disease transmission, we implemented a stochastic SEIR (Susceptible–Exposed–Infectious–Recovered) (Figure S2) model augmented with an explicit environmental compartment to simulate outbreak dynamics for two disease systems with environmental transmission and contrasting life histories: Influenza A virus and swine brucellosis (Brouwer et al. 2017; Ghosh et al. 2023). This enhanced model captures indirect transmission via environmental reservoirs, thereby allowing us to explore how differences in transmission mechanisms and network structures influence key epidemiological outcomes such as disease persistence, epidemic peaks, and the basic reproduction number (*R*_*0*_) (Ogbunugafor et al. 2020). Details of the SEIR model and their structures can be found in Table S2.

### 2.3 Model Evaluation and Analysis

To contrast the effect of different assumptions about environmentally mediated contact on transmission dynamics, we used a structured assessment framework to approximate the total dyadic contacts and count of unique host pairs identified by each contact model for each pathogen persistence scenario (7‐day and 30‐day windows). We also monitored the maximum frequency of contact between each pair of individuals throughout the whole 259‐day observation period to enable heterogeneity in exposure intensity.

Second, we constructed weighted, directed contact networks from the estimated contact matrices of each model. Each pair is unidirectional and unique, that is, A → B is not equal to B → A. To summarize the structural characteristics of such networks, we calculated four widely used measures with epidemiological relevance like edge density, which is the fraction of observed contacts relative to all possible dyads; transitivity or global clustering coefficient, which explains the tendency of nodes to get connected with densely connected triads that may sustain local outbreaks; modularity, which measures the degree to which networks are divided into distinct communities that may act as transmission bottlenecks; and degree assortativity, which measures the correlation in node connectivity and may influence epidemic spread velocity and amplitude. We also generated spatial risk surfaces for each of these models by rastering contact intensity onto the 30 × 30 m grid cells used by the behavioral and high‐use models. This allowed us to see and compare the spatial pattern of transmission‐relevant contact hotspots under each of the definitions of contacts.

## 3 Results

### 3.1 Contact Kernels

**FIGURE 2:**
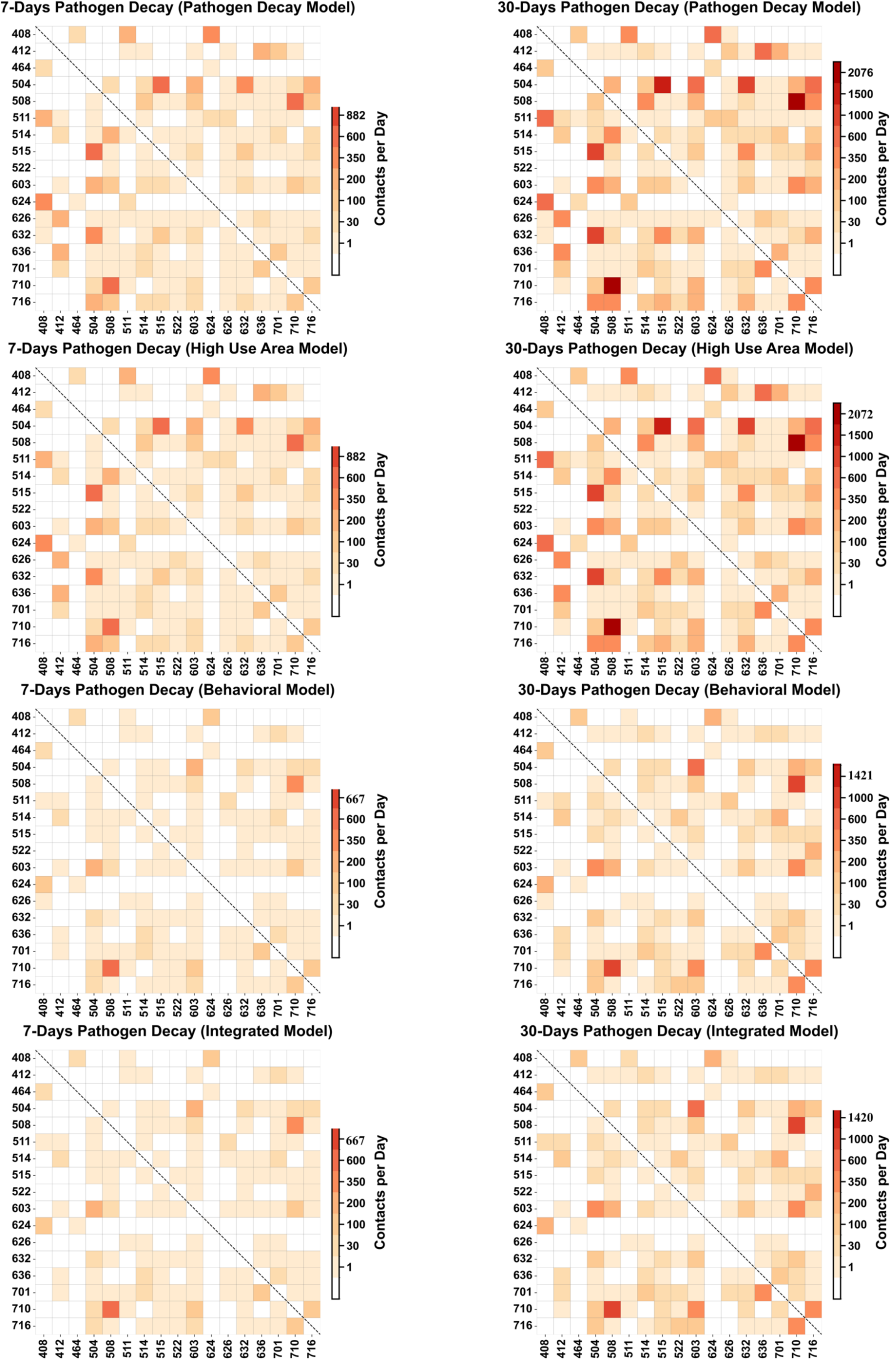
Contact Kernel plots showing the total number of indirect contacts between each pair of wild pigs under four modeling for both 7‐day and 30‐day pathogen decay. The *x*‐ and *y*‐axes indicate the unique animal IDs, with each cell representing the cumulative contact count between a pair. Darker colors correspond to a higher number of contacts.

The pathogen decay model recorded the highest number of unique pairs with 162 for a 7‐day pathogen persistence scenario and 176 for a 30‐day scenario out of a possible 256 unique pairs, alongside maximum contact counts of 882 and 2075 across 259 days, respectively (Figure 2). In the high‐use area model, while the maximum contact counts remained similar (882 for the 7‐day scenario and 2072 for the 30‐day scenario), the unique pairs slightly decreased to 159 for the 7‐day scenario and 172 for the 30‐day scenario. As expected, the behavioral model showed a marked reduction in both contact counts and unique pairs (because some of the movement behaviors had 0 probability of transmission as defined through the step function), with maximum counts decreasing to 667 for 7 days and 1421 for 30 days, and unique pairs reducing to 136 for 7 days and 149 for 30 days. The integrated model had the lowest unique pairs, with 135 for 7 days and 148 for 30 days, and maximum contact counts of 666 for 7 days and 1420 for 30 days. These results illustrate a consistent reduction in unique pairs and contact counts across models as stricter criteria were applied. For the 7‐day decay model, a shift from the pathogen decay model to the high‐use model led to a moderate 0.30% decline in total contacts. Conversely, there were marked declines against the behavioral (70.34%) and the integrated (70.43%) models. As indicated, such tendencies were replicated under the 30‐day decay model, showing consistent model‐driven variation in the reduction of contact (Figure S3).

### 3.2 Network Structure and Matrices

**FIGURE 3:**
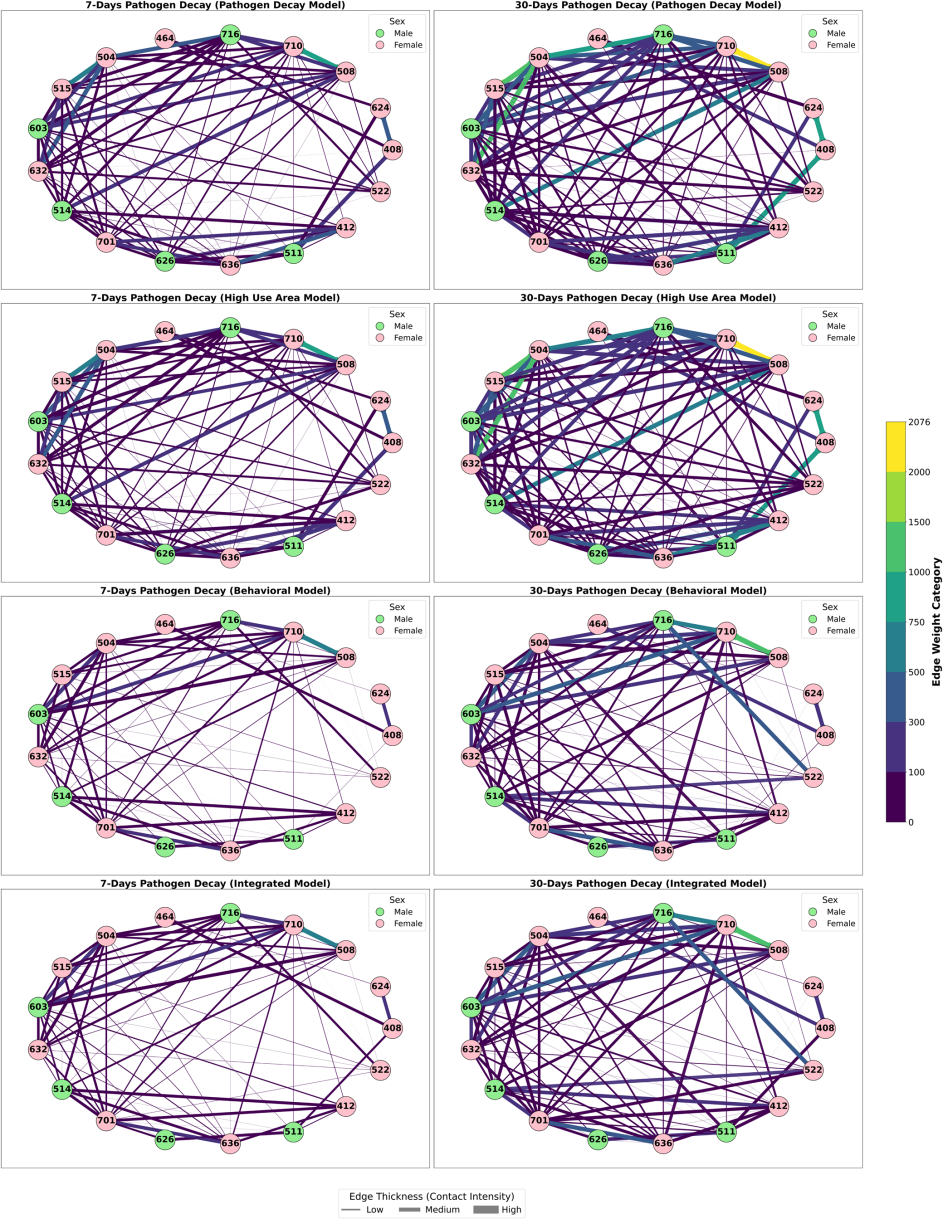
Contact network structure under different contact models for 7‐day and 30‐day pathogen decay scenarios. Each node represents an individual wild pig, and each edge denotes the amount of contacts. Edge thickness and color intensity correspond to the number of indirect contacts.

The network structure and corresponding matrices (Figure 3 and Table S1) reveal differences in clustering and connectivity across models. The pathogen decay model shows the highest edge density (0.60 for 7 days, 0.65 for 30 days) and strong local clustering with transitivity values of 0.81 and 0.84. Modularity is moderately high (0.50 for 7 days, 0.44 for 30 days), while assortativity is low (0.29 and 0.17), indicating a network with widespread connectivity and weak degree assortative patterns. The high‐use area model increases modularity slightly (0.52 for 7 days, 0.46 for 30 days) and achieves the highest assortativity (0.43 for 7 days, 0.30 for 30 days), highlighting more structured clustering. The behavioral and integrated models both show the same edge density (0.50) for 7 days and slightly different values for 30 days, with the same modularity of 0.42 for 7 days and 0.37 for 30 days in both models. Transitivity stays at 0.77–0.79, and assortativity declines to its lowest values (0.26 for 7 days, 0.30 for 30 days), reflecting sparse, selectively filtered networks.

### 3.3 Spatial Distribution

**FIGURE 4:**
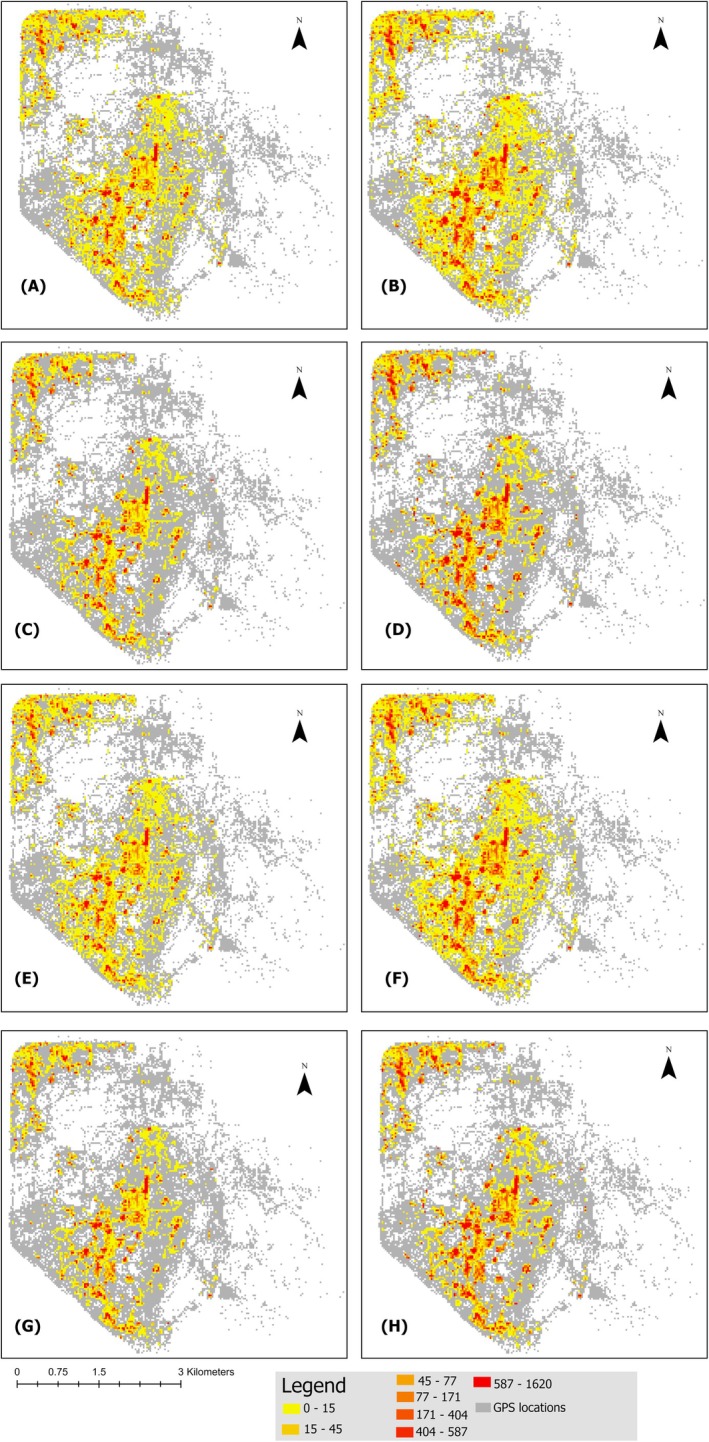
Spatial distribution of contact in landscape under different models; (A) pathogen decay only (7 days), (B) pathogen decay only (30 days), (C) high use area (7 days), (D) high use area (7 days), (E) behavioral model (7 days), (F) behavioral model (7 days), (G) Integrated model (7 days), (H) Integrated model (30 days). Grid cells represent 30 × 30 m spatial units. Gray areas indicate zones with no contact, yellow indicates locations where contacts occurred, and red denotes high‐intensity contact zones.

We define “contact zones” as areas where contact events occur, and “high‐intensity areas” as contact zones with the highest contact frequencies. The pathogen decay model displays widespread contact zones (yellow colored zone in Figure 4), with high‐intensity areas (red colored zone in Figure 4) concentrated in central and peripheral regions for both 7‐ and 30‐day durations (Figure 4). The high‐use area model reduces the extent of these zones, concentrating high‐intensity areas in revisited central patches, while peripheral regions become less prominent. In contrast, the behavioral model reveals a pattern similar to the pathogen decay model, with widespread but slightly more localized contact zones, as transient activities are filtered. The integrated model produces the most selective pattern, confining high‐intensity areas to a few small, central patches where spatial and behavioral criteria overlap. The distribution narrows progressively in the high‐use and integrated models, while pathogen decay and behavioral models retain broader spatial patterns. Pairwise differences and spatial distributions of contacts among different models have been shown in Figures S8 and S9.

### 3.4 Disease Transmission

**FIGURE 5:**
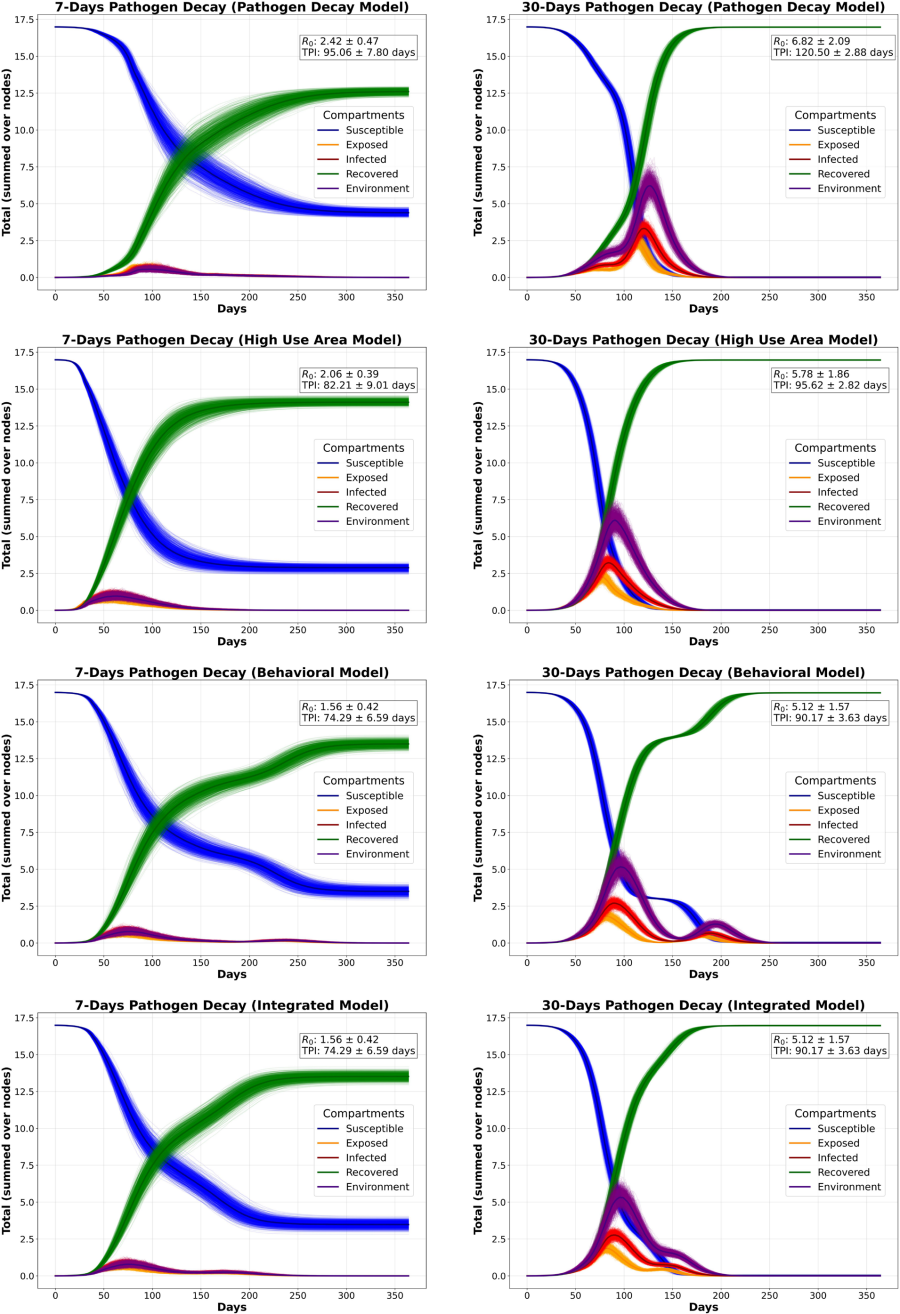
SEIR epidemic simulations for 7‐day and 30‐day pathogen decay durations under different contact modeling scenarios. For a specific contact model, each panel shows the mean trajectories (of 1000 runs) of the epidemiological compartments (Susceptible, Exposed, Infected, Recovered, and Environmental Load) over a period of 365 days.

The SEIR model outputs illustrate how extending pathogen persistence from 7 to 30 days fundamentally alters the epidemic curve and the probability of transmission (Figure S4) in each environmentally mediated contact model. For the 7‐day scenarios, the Pathogen Decay model carries the highest *R*_*0*_ of 2.42 ± 0.47 (mean ± standard deviation) and peaks in incidence at 95.06 ± 7.80 days, while the High‐use, Behavioral, and Integrated models have successively lower *R*_*0*_ values (2.06 ± 0.39, 1.56 ± 0.42, and 1.35 ± 0.50, respectively) with peak incidence at 82.21 ± 9.01–74.73 ± 6.67 days (Figure 5). Under 30‐day persistence, *R*_*0*_ rises steeply in the Pathogen Decay model to 6.82 ± 2.09 with a delay in peak incidence to 120.50 ± 2.88 days, whereas the other models are of the same trend with *R*_*0*_ 5.12 ± 1.57 (Behavioral) to 4.62 ± 1.60 (Integrated) with time to peak incidence decreasing to 89.87 ± 3.37 days.

## 4 Discussion

Environmentally mediated contacts play a critical role in disease transmission dynamics for environmentally resilient pathogens such as *Bacillus anthracis* , *Brucella* spp., and influenza A viruses by enabling pathogens to persist in the environment and facilitating indirect transmission even when hosts are present simultaneously. Previously, researchers often defined those contacts based on colocation of hosts within a segregated timeframe, which can overestimate the environmentally mediated contacts, as not all instances are epidemiologically relevant. Building on a previous framework, this paper offers a refined framework for quantitatively defining relevant indirect contacts by considering pathogen decay, host movement, and behavior. We applied this framework to wild pigs in Florida to demonstrate how pathogen persistence, high‐use areas, and host behavioral states shape the environmentally mediated contact networks. We found that while prolonged pathogen survival enhances connectivity, the contamination levels and host behaviors restrict transmission risk in specific ways. Our model framework captures environmentally mediated contacts via the environment at different levels of epidemiological relevance, providing a nuanced understanding of disease transmission.

### 4.1 Tradeoffs of Different Model Structures

Our framework presents four alternative structures for characterizing environmentally mediated contacts, each of which embodies different assumptions about host behavior, space use, and pathogen viability. By articulating these models and comparing them, we illustrate how transmission estimates and the contact networks that support them, are sensitive to the definition of epidemiologically relevant exposure. This comparative framework is one of the strengths of our approach: it allows us to identify which assumptions have the greatest effect on transmission risk and which are scenario‐consistent.

Although the pathogen decay model is straightforward to apply, it is often oversimplified in many real‐world disease systems, since pathogen decay is not the only factor determining transmission. Pathogen deposition and acquisition both influence transmission, and ideally, we would consider behaviors related to these processes in the identification of relevant environmentally mediated contacts. Some behavioral states, such as resting and foraging states, which have long durations and high visitation, respectively, are often considered as having high potential for pathogen deposition and acquisition. However, these states do not necessarily represent specific behaviors or actions (Pohle et al. 2017). The detection of certain deposition and acquisition behaviors, such as urination and defecation, cannot rely on host movement data only and often requires additional monitoring methods, such as camera traps (Egan et al. 2023). Camera traps and video footage from individuals wearing video devices can supply useful validation information for behavioral state assignments in movement models, although they only record a very limited temporal window of animal activity (Lavelle et al. 2012, 2014). Camera trap detections that overlap with GPS‐collared animals could be used to estimate within‐state rates of behavioral activity and test state‐specific activities (e.g., resting or foraging), which can improve interpretation and parameterization of state‐dependent contact models. The high‐use area model in our framework, which considers the pathogen deposition risk as high visitation areas, can be used to capture the proxy of deposition/acquisition, as it identifies where repeated host revisitation increases exposure risk. However, this approach may not capture the full extent of transmission that is occurring outside of the pre‐defined highly concentrated areas. Some new technologies combining GPS collars with cameras are developing and can be used to better understand movement and behavior in the context of these models (Lavelle et al. 2012, 2014).

Although the behavioral model excludes contact during movement states to avoid overestimation of exposure risk, animals may still deposit pathogens during movement. Excluding this behavior can lead to underestimation of contamination risk for frequently traveled movement corridors. Many species repeatedly use the same corridors to transit between habitats, increasing the risk of contamination and pathogen exposure along those routes. The presented integrated model, which both accounts for acquisition behaviors and incorporates the high‐use areas as deposition sites, can retain such corridor‐based contacts. This is particularly important for species with high site fidelity, where repeated movement patterns maximize the likelihood of exposure. Our results indicate that behaviorally bounded contact networks can generate slower or delayed epidemic peaks, capturing dynamics that previous methods were unable to resolve.

Our models can also be applied to forecast hotspots of transmission through the integration of predictive movement modeling and environmental data. Future trajectories of individual animals can be predicted using predictive movement models, such as step selection functions (Thurfjell et al. 2014; Potts et al. 2022; Potts and Börger 2023) or continuous‐time movement models (CTMM; Codling et al. 2008), parameterized on the basis of historical movement behavior and environmental covariates. The predicted trajectories can be used to project the spatiotemporal overlap of contaminated patches and host presence when combined with pathogen environmental persistence models. In those systems where upcoming host‐pathogen overlap would tend to be foreseeable, that is, in those with migratory behavior, site fidelity, memory or seasonal movement patterns, this approach is particularly well‐suited. The applicability of the everyday movement model and presence of representative training data, however, determine how reliable such projections are.

### 4.2 Insights From the Case Study

Wild pigs are a group‐living species with a complex social structure, typically forming matriarchal groups consisting of females and their offspring. This social organization results in high contact rates within social groups, while contact rates between groups are relatively low. However, between‐group contacts are often the primary drivers of disease spread. In our case study, which involved collaring wild pigs from different groups, our models indicate that even between‐group contacts play a disproportionately large role in transmission, particularly those contacts filtered by space and behavior. While less common, intergroup contacts are important bridging ties that link highly connected within‐group clusters, improving overall network connectivity and allowing for wider transmission throughout the population. In particular, high weighted indirect contact pairs seem to often indicate transient group merging or range overlap, and fission–fusion processes therefore influence not only contact structure but also potential outbreak pathways (Yang, Proffitt, et al. 2021; Yang, Schlichting, et al. 2021).

When implemented to contrasting pathogens (IAV and *Brucella suis* ), our method reveals that assumptions about contact relevance make significant impacts on epidemic projections. For instance, including all spatial intersections without behavioral or temporal filtering leads to overestimation of *R*_*0*_, especially for very long‐lived pathogens like *Brucella suis* (Pinn‐Woodcock et al. 2023; Pepin et al. 2023). Conversely, behavioral filter use leads to lower *R*_*0*_ values and epidemics with later peaks that better reflect empirical ecological data (Hoyt et al. 2023; Pepin et al. 2021). Because acquisition of pathogens can only occur during particular host activities (i.e., foraging and resting in our application), the behavioral model delayed and flattened SEIR curves through reducing opportunity for successful transmission after deposition had taken place. Our comparative findings reflect how host behavior, visitation patterns, and pathogen breakdown influence each other to produce indirect contact risk, which is extremely context‐sensitive (Dougherty et al. 2022; Rees et al. 2021). Traditional models frequently obscure this variability, highlighting the value of integrative, system‐specific frameworks for comprehending the transmission of diseases mediated by the environment. Our method has roots in earlier modeling of indirect contact (i.e., Yang et al. 2023; Wilber et al. 2022; Dougherty et al. 2018), which customarily assigns equal epidemiological relevancy to all the indirect overlaps. In contrast, we illustrate how behavior state and visitation intensity alter the expected risk landscape in a way consistent with the experienced pathogen biology in systems such as brucellosis or IAV.

### 4.3 Extensions, Future Directions, and Limitations

#### 4.3.1 Extensions and Future Directions of the Models

To illustrate the implementation of our modeling framework for environmentally mediated contact, we employed a single species system as a case study. Similar concepts have been shown in multi‐host systems (Herraiz, Triguero‐Ocaña, et al. 2024), but our approach is novel in its modular design, explicitly separating and contrasting mechanistic processes like environmental degradation, state‐of‐behavior filtering, and high‐usage area identification to estimate how each affects indirect contact networks and epidemic outcomes. Our models can be extended to include more sophisticated ecological settings, such as environmentally persistent or vector‐borne multi‐host pathogens. Multi‐host disease systems involve species with varying movement patterns, habitat use, and susceptibility, all of which influence indirect transmission risk via the environment (Webster et al. 2017; Lambin et al. 2010). Unlike earlier models that treat all indirect contact as epidemiologically equivalent, our model allows researchers to specify species‐ or behavior‐specific causes of environmental contamination and risk (Murray et al. 2016; Titcomb et al. 2021). For example, in the brucellosis system with the elk–cattle interface, our framework can help identify the core shedders and the acquirers based on their behaviors or space‐use patterns as well as their transmission hotspots (Berry and Wells 2018). Expanding the framework to a multi‐species context would also involve integrating species‐specific movement and behavioral data as well as pathogen compatibility across host species. Further studies have shown that species interactions, for example through competitive exclusion or habitat partitioning may modify environmentally mediated contact structures that affect disease transmission dynamics (Baishya et al. 2021). Incorporating these variations would enhance the predictive power of environmentally mediated contact models and provide more accurate estimates of interspecies transmission risk.

Additionally, although our framework is designed explicitly for quantifying environmentally mediated contact, it also has the potential to be extended to a vector‐borne system, which is considered challenging from an epidemiological perspective because vectors act as both pathogen reservoirs and transmission agents. Vector‐borne diseases are distinct from environmentally mediated transmission because pathogen decay occurs throughout both environmental transmission and the vector itself (Khong et al. 2023) and most vectors can move (thus the point source changes location). For example, in tick‐borne disease systems, a tick can pick up a pathogen from one host and then remain infected for an extended period before transmitting the pathogen to another host during its subsequent feeding (Boulanger et al. 2019). However, not all tick‐host interactions are followed by transmission since vector competence alongside pathogen replication within the vector and host immune responses determines the probability of transmission success (Rocha et al. 2022). The integration of vector distribution, their biting rate, and habitat use into the framework will provide better estimates of when and where transmission events are most likely to occur. Furthermore, environmental factors including temperature and humidity which influence both tick activity and pathogen viability need to be included to enhance transmission risk predictions (Rocha et al. 2022). In this context, vector‐related processes can be integrated through the host‐contact and environmental persistence modules, which allow for the explicit definition of parameters like habitat preference, biting rate, and vector density. Furthermore, to better capture the dynamics of vector‐mediated transmission, environmental modifiers (such as temperature and humidity) that affect vector activity and pathogen viability can be included in the decay and exposure functions. This enables evaluation of where and when spillovers are most likely, particularly under climate‐sensitive circumstances that change vector distribution and activity windows.

The inclusion of temporally and spatially variable environmental factors that are known to affect pathogen persistence, such as temperature, moisture, pH, soil type, and solar radiation, is an important extension of this framework. Even though our current implementation assumes simple exponential decay, persistence is frequently nonlinear under various environmental conditions. For instance, depending on soil chemistry and organic matter content, the prions of chronic wasting disease can remain infectious for months or years (Otero et al. 2021). Future models would be more representative of real‐world heterogeneity if covariates like decay modifiers were included (Wißmann et al. 2021; Cohen et al. 2020). This would also enhance forecasts for pathogens like influenza, whose stability varies seasonally with climate. Building on earlier frameworks (Wilber et al. 2022), this refinement would enable mechanistic testing of how environmental variability alters indirect transmission risk across host–pathogen systems.

#### 4.3.2 Limitations of the Case Study

There are also some limitations within the case study. First, the model presumes equal host susceptibility, but immune response variability or prior exposure likely affects acquisition risk (Siva‐Jothy and Vale 2021). Future versions should consider immunodynamics and heterogeneity to improve transmission estimates. Second, accurately parameterizing shedding and acquisition within environmental compartments remains challenging due to limited empirical data on infectious doses and host behaviors (Pepin et al. 2023; Brouwer et al. 2017). While the behavioral module herein uses a simple binary filter, this modular framework supports the addition of probabilistic weighting of network edges, accelerometer‐informed classification of contacts, and habitat‐based refinements to more fully represent behavioral effects on transmission (Egan et al. 2023; Triguero‐Ocaña et al. 2021). Finally, while we aim for high‐use areas as a deposition surrogate, landscape heterogeneity (e.g., vegetation cover, water, resource distribution) likely influences both host movement and pathogen survival. The incorporation of such environmental factors in future models will enhance the predictive mapping of spatiotemporal risk (Bradley and Lockaby 2023; Esposito et al. 2023).

## 5 Conclusions

This study offers a flexible framework for modeling environmentally mediated transmission in a variety of ecological contexts by incorporating behavioral state, intensity of space use, and environmental viability within a shared framework. We illustrated the applicability of the model through case studies utilizing GPS tracking data from feral pigs. Our case study results found that contact network topology for indirect contact and the predicted pathogen spread pattern are sensitive to assumptions about host behavior, spatial overlap, and pathogen persistence in the environment. Inflated *R*_*0*_ and distorted epidemic dynamics can lead to models that disregard behavioral context or overextend the duration of exposure relevance. These results highlight the need to create epidemiologically relevant definitions of indirect contact that are consistent with both host ecology and pathogen biology. By operationalizing deposition, persistence, and acquisition processes explicitly, this framework offers a clear tool for making mechanistic hypotheses about indirect transmission. Furthermore, the modular design facilitates future extensions to multi‐host and vector‐borne systems, incorporation of environmental covariates such as land cover or climatic variables, and refinement using higher‐resolution behavioral data (e.g., accelerometry). Consequently, this framework not only improves the interpretability of system‐specific dynamics but also helps address the larger objective of building generalizable, comparative insights into the role of environmental processes in transmission across host–pathogen systems.

## Author Contributions

**Himel Talukder:** conceptualization (equal), data curation (equal), formal analysis (equal), investigation (equal), methodology (equal), software (equal), writing – original draft (equal), writing – review and editing (equal). **Ryan S. Miller:** methodology (equal), supervision (equal), writing – review and editing (equal). **Raoul K. Boughton:** data curation (equal), methodology (equal), writing – review and editing (equal). **Kurt C. Vercauteren:** methodology (equal), writing – review and editing (equal). **George Wittemyer:** writing – review and editing (equal). **Kim M. Pepin:** methodology (equal), writing – review and editing (equal). **Anni Yang:** conceptualization (equal), data curation (equal), methodology (equal), supervision (equal), writing – original draft (equal), writing – review and editing (equal).

## Funding

This work was supported by the National Wildlife Research Center and Research Corporation for Science Advancement.

## Ethics Statement

The animal study and capture protocol was approved by the University of Florida Institutional Animal Care and Use Committee (IACUC #201408495 and # 201808495). The study was conducted in accordance with local legislation and institutional requirements.

## Conflicts of Interest

The authors declare no conflicts of interest.

## Supporting information


**Appendix S1:** ece372571‐sup‐0001‐AppendixS1.docx.

## Data Availability

All the required data is publicly available in figshare repository: https://figshare.com/articles/dataset/_b_Estimating_Environmental_Transmission_Risk_from_Host_Movement_Data_b_/29905793?file=57173513.
