# Supplementary material for: Estimating Environmental Transmission Risk From Host Movement Data
Source: Ecol Evol. 2025 Nov 30;15(12):e72571. doi: 10.1002/ece3.72571 (PMC12665434; doi:10.1002/ece3.72571)
Supplement: Supplementary file 1 — Appendix S1: ece372571‐sup‐0001‐AppendixS1.docx. [file ECE3-15-e72571-s001.docx]

***Supporting information for :***

**Estimating Environmental Transmission Risk from Host Movement Data**


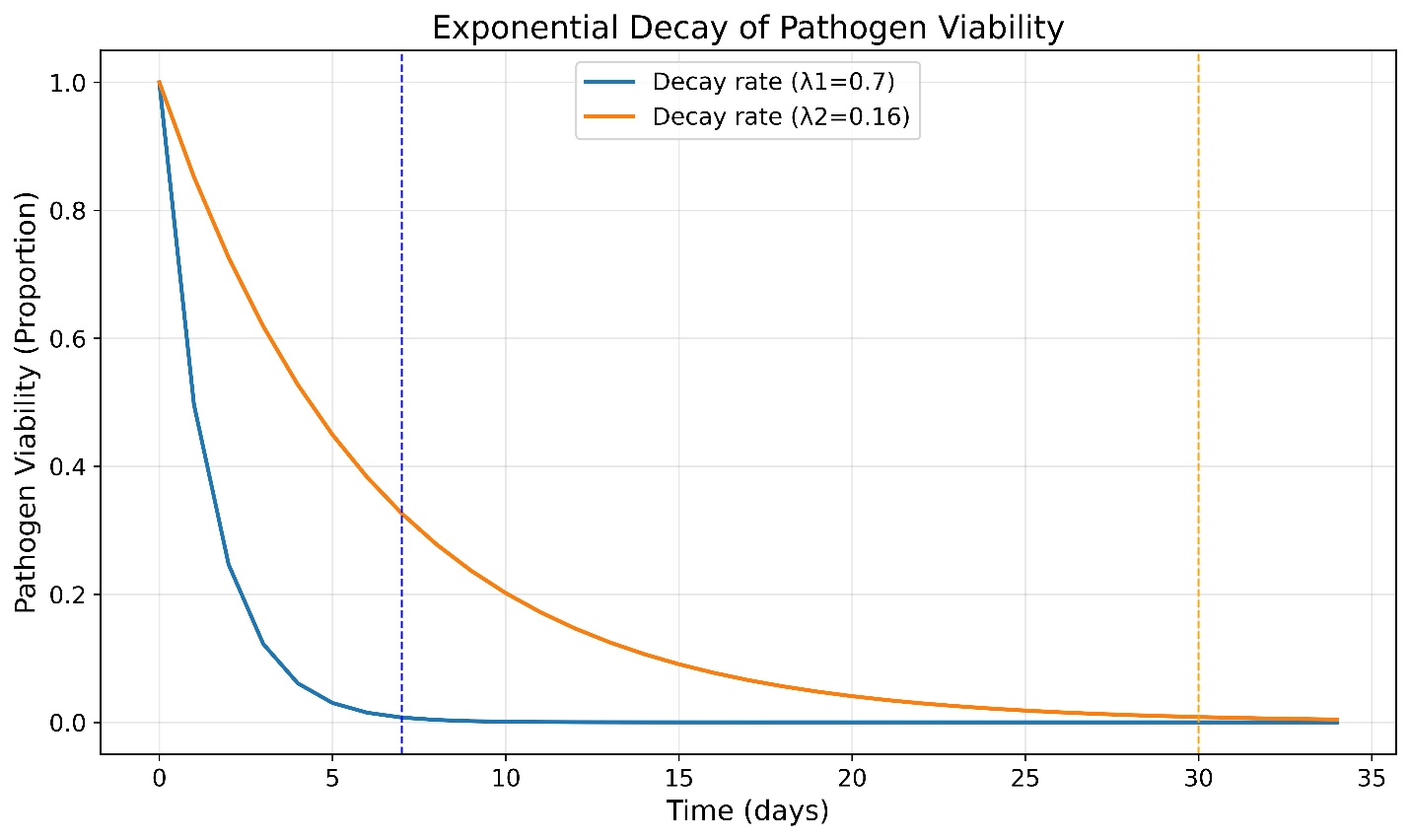


Figure S1. Pathogen viability with exponential decay for two survival windows (Dashed lines showing the days at which pathogen load in the environment becomes zero).

Table S1. Network matrices among 4 different models in 7-day and 30-day pathogen decay

|  | **Pathogen decay model** | | **High-use area model** | | **Behavioral model** | | **Integrated model** | |
| --- | --- | --- | --- | --- | --- | --- | --- | --- |
| **Matrices** ​ | **Indirect (7 days)** ​ | **Indirect (30 days)** ​ | **Indirect (7 days)** ​ | **Indirect (30 days)** ​ | **Indirect (7 days)** ​ | **Indirect (30 days)** ​ | **Indirect (7 days)** ​ | **Indirect (30 days)** ​ |
| Modularity ​ | 0.50​ | 0.44​ | 0.52​ | 0.46​ | 0.42​ | 0.37​ | 0.42 | 0.37 |
| Transitivity ​ | 0.81 ​ | 0.84​ | 0.85​ | 0.87​ | 0.77​ | 0.79​ | 0.77 | 0.79 |
| Edge Density ​ | 0.60​ | 0.65​ | 0.58​ | 0.63​ | 0.50​ | 0.55​ | 0.50 | 0.54 |
| Assortativity ​ | 0.29 ​ | 0.17​ | 0.43​ | 0.30​ | 0.27​ | 0.31​ | 0.26 | 0.30 |
| Outdegree ​ | norm (μ =9.53, σ = 3.63), x = [2,15]​ | norm (μ =10.35, σ = 3.58), x = [2,15]​ | norm (μ =9.35, σ = 3.44), x = [2, 13]​ | norm (μ =10.12, σ = 3.36), x = [2, 14]​ | norm (μ =8, σ = 3.24), x = [2, 13]​ | norm (μ =8.76, σ = 3.32), x = [2, 13]​ | norm (μ =7.94, σ = 3.28), x = [2, 13]​ | norm (μ =8.71, σ = 3.37), x = [2, 13]​ |
| Indegree ​ | norm (μ =9.53, σ = 3.24), x = [2,13]​ | Gamma (α= 234.68, β= 0.23), x = [3,15]​ | norm (μ =9.35, σ = 3.53), x = [2, 13]​ | norm (μ =10.12, σ = 3.82), x = [3, 15]​ | norm (μ =8, σ = 3.33), x = [2, 13]​ | expon (λ = 2), x = [2,13]​ | norm (μ =7.94, σ = 3.40), x = [2, 13]​ | Gamma (α= 271.96, β= 0.21), x = [2,13]​ |
| Outstrength ​ | Lognorm (α= 0.40, β = 1035.58) ​x = [49.64,1886]​ | Lognorm (α= 0.46, β = 2396.48) ​x = [148.19,5506.52]​ | Lognorm (α = 0.40, β = 1032.70), x = [64.92, 1921.07]​ | Lognorm (α =0.46, β = 2363.73), x = [212.36, 5542.75]​ | expon (λ = 13.85), x = [13.85, 1084.24]​ | Lognorm (α =0.80, β = 620.02), x = [22.09, 2722.64]​ | expon (λ = 13.56), x = [13.56, 1093.69]​ | Lognorm (α =0.79, β = 645.17), x = [21.58, 2752.60]​ |
| Instrength ​ | norm (μ =689.81, σ = 429.105), ​x = [42.89,1527.19]​ | Lognorm (α= 0.35, β = 3256.64) ​x = [140.28,3946.87]​ | norm (μ =702.53, σ = 428.17), x = [90.78, 1541.65]​ | Lognorm (α = 0.43, β = 2566.81), x = [257.17, 3961.40]​ | expon (λ = 3.40), x = [3.40, 1031.91]​ | expon (λ = 15.64), x = [15.64, 3112.58]​ | expon (λ = 18.26), x = [18.26, 1039.28]​ | expon (λ = 78.52), x = [78.52, 3139.28]​ |
| Eigenvector​ (mean) | 0.16 | 0.15​ | 0.16​ | 0.16​ | 0.12​ | 0.13​ | 0.12 | 0.13 |
| Closeness​(mean) | 0.67 | 0.72 | 0.64​ | 0.71​ | 0.58 | 0.62 | 0.58 | 0.62 |
| Betweenness​(mean) | 0.17 | 0.16​ | 0.14​ | 0.12​ | 0.16 | 0.17 | 0.15 | 0.15 |


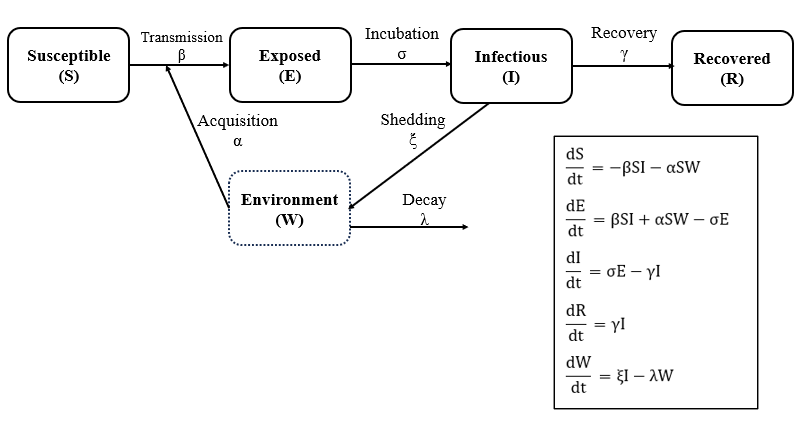


Figure S2: Schematic Diagram of the environmentally mediated SEIR model with their respective PDEs. Parameters are defined in table S2.

**SEIR Model Specifications**

We parameterize the SEIR epidemic model to properly assess disease-specific transmission dynamics and provide realistic simulation of outbreak progression. Adequate parameterization enables the model to mimic biological processes including incubation periods, infectious periods, and contact rates. The environmental compartment explicitly models pathogen accumulation through shedding from infected individuals, its subsequent decay in the external environment, , and eventual acquisition by susceptible hosts (Brouwer et al., 2017). Parameterization reflects the distinct biological properties of the pathogens : Influenza A virus exhibits rapid decay in external environments while *Brucella suis* shows prolonged environmental stability in contaminated water and soil (Buch et al., 2023). Stochastic transmission (β), incubation (σ), recovery (γ), shedding (ξ), and environmental acquisition (α) rates capture natural infection heterogeneity at host and environmental interfaces (Table S2), according to recent empirical research (Antolin, 2008; Buch et al., 2023). Deterministic pathogen decay rates (λ) capture differential rapid versus long-term environmental survival, as a function of exposure duration, according to pathogen longevity traits. Initial infection proportion (I₀) was initialized to simulate initial outbreak seeding in controlled environments. In our simulations, we conducted 1,000 stochastic iterations over a 365-day period per run, thereby capturing natural variability and enabling a robust assessment of how environmental persistence interacts with contact network characteristics to drive transmission dynamics (Dureau et al., 2013).

Key epidemiological metrics, including R₀ and time to peak incidence, were estimated from the simulation outcomes. By integrating these metrics, along with extinction probabilities where applicable, our model provides a comprehensive framework to evaluate how variations in contact structures and environmental transmission pathways influence the spread and persistence of disease within a population.

Table S2- Parameters used in the SEIR disease transmission model

| **Parameter** | **Short-lived (7 days)** | **Long-lived (30 days)** | **Type** | **Reference** |
| --- | --- | --- | --- | --- |
| Transmission Rate (β) | 0.1 - 0.3 | 0.05 - 0.2 | Stochastic | Antolin, 2008; Buch et al., 2023 |
| Incubation Rate (σ) | 0.13 - 0.30 | 0.05 - 0.1 | Stochastic | Virlogeux et al., 2015; Elmonir et al., 2022 |
| Recovery Rate (γ) | 0.1 - 0.2 | 0.07-0.14 | Stochastic | Mu et al., 2018; Elmonir et al., 2022 |
| Pathogen Decay Rate (λ) | 0.7 | 0.16 | Deterministic | User defined |
| Environmental Shedding Rate (ξ) | 0.3 - 0.6 | 0.2 - 0.4 | Stochastic | Bhattarai et al., 2011; Rebollada-Merino et al., 2022 |
| Environmental Acquisition Rate(α) | 0.2 - 0.5 | 0.2 - 0.4 | Stochastic | Brouwer et al., 2022; Rebollada-Merino et al., 2022 |
| Initial Proportion Infected (I_o_) | 0.01 - 0.03 | 0.01 - 0.03 | Deterministic | User defined |


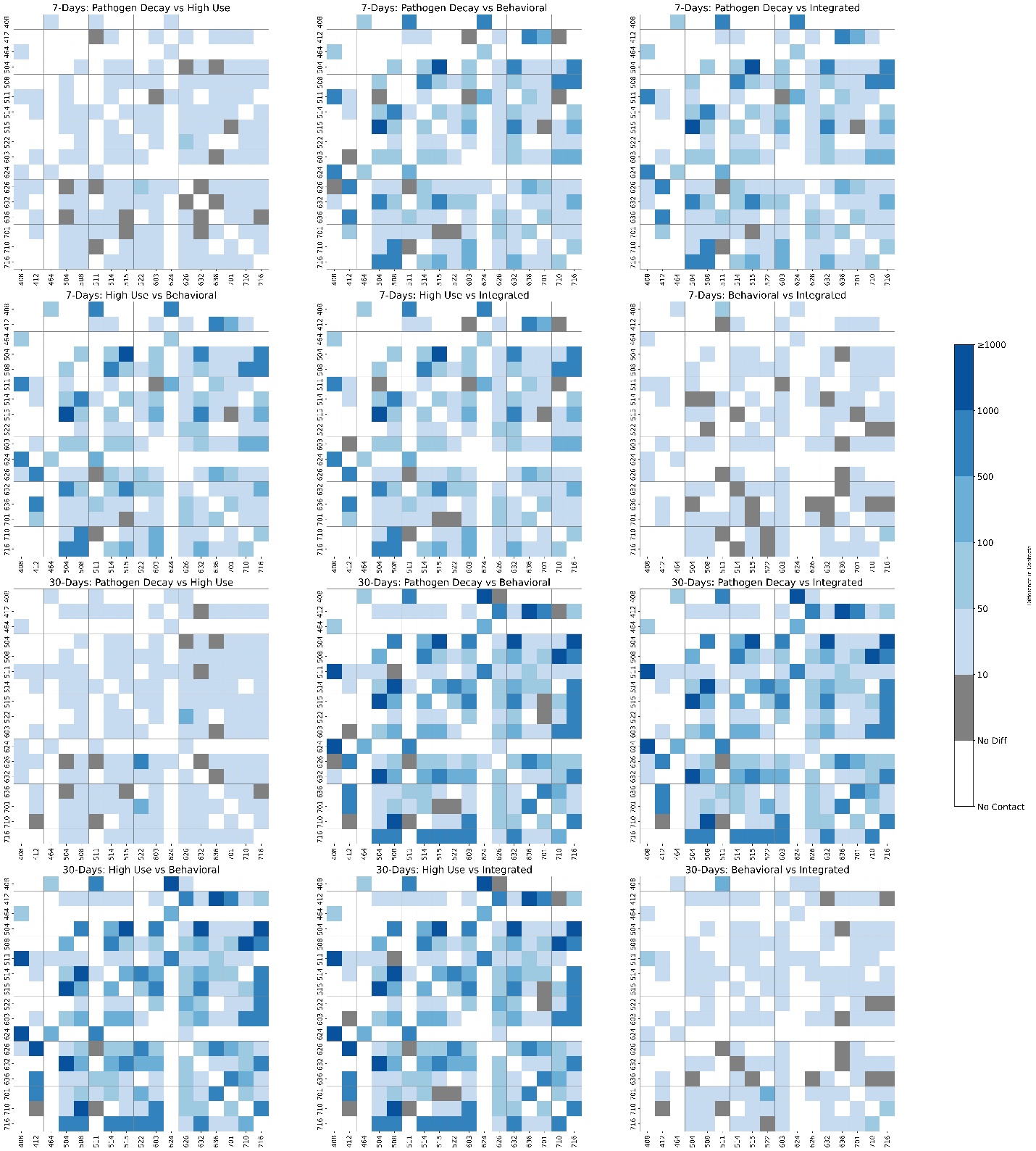


Figure S3- Pairwise comparison of the difference of contact between different models.


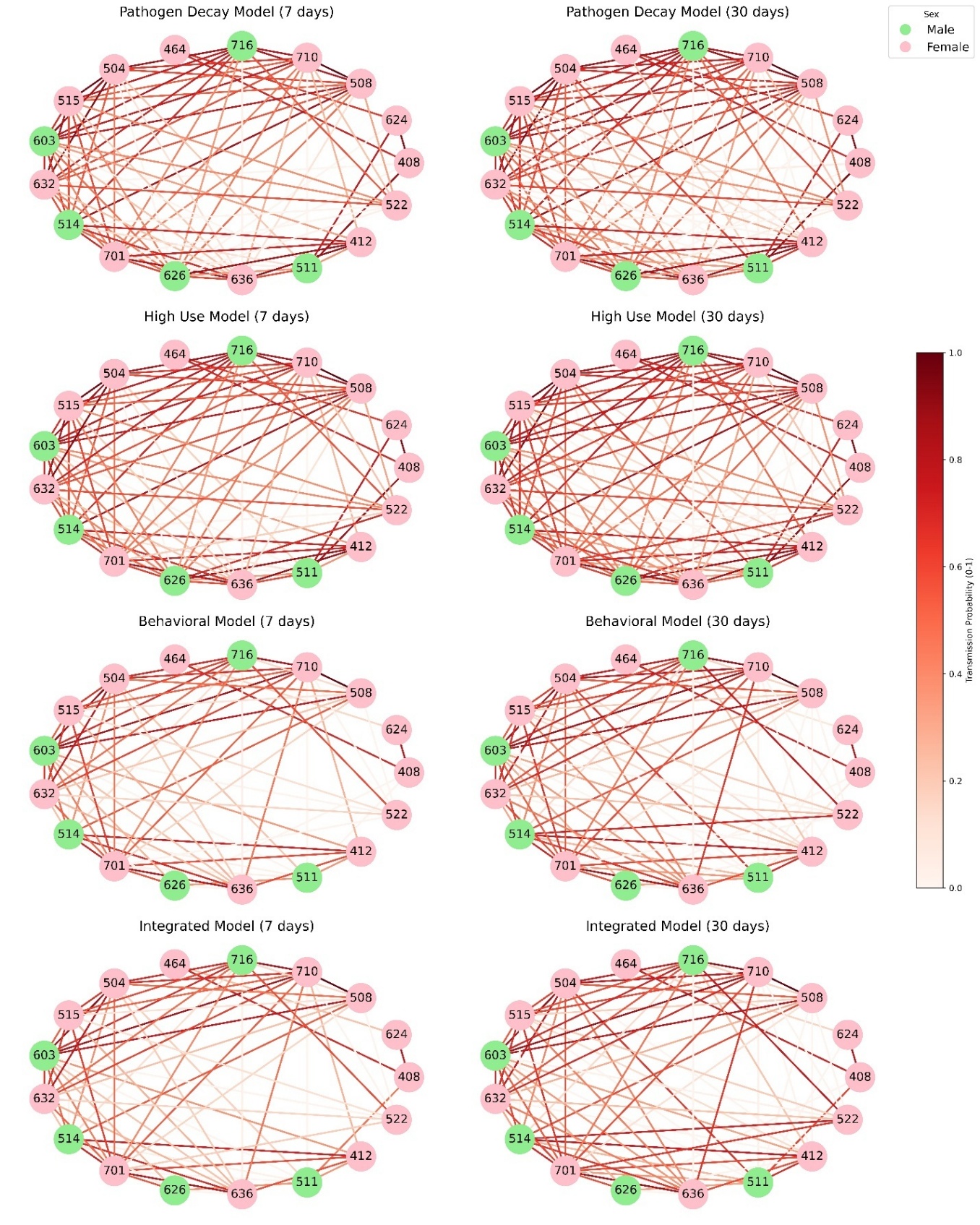


Figure S4- Probability of transmission network, based on the SEIR model.

**Sensitivity Analysis for high-use area:**

We have tested the 50 and 90 percentiles to define the high-use area and how these thresholds influence the identification of relevant environmentally-mediated contacts.


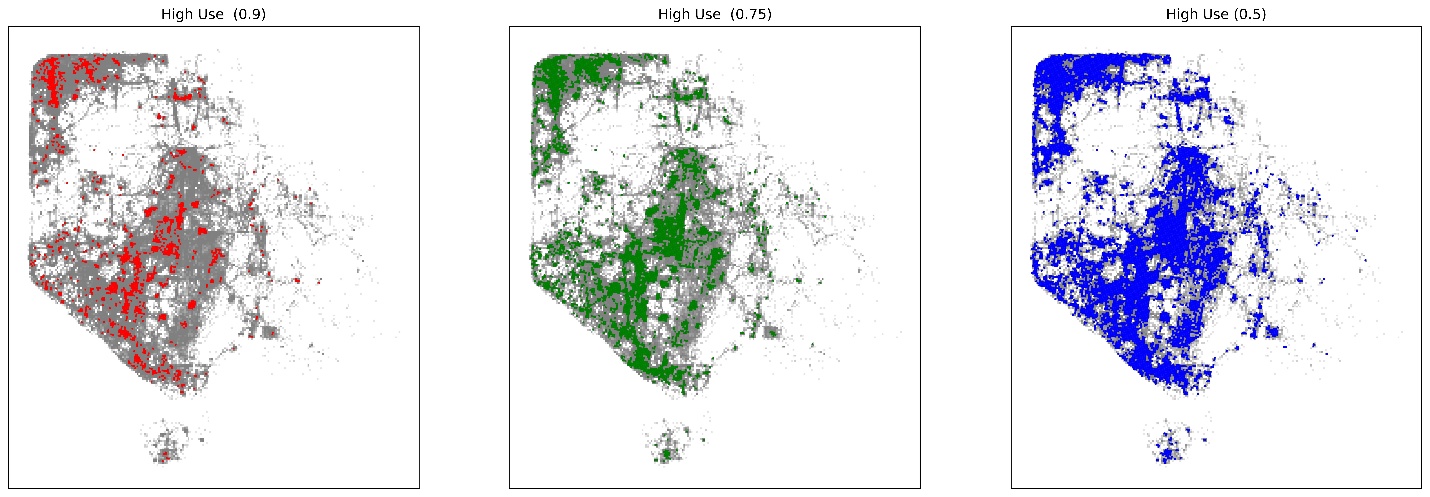


Figure S5 – High-use areas under different definitions oh visitation rates with 50^th^ , 75^th^ and 90^th^ percentile


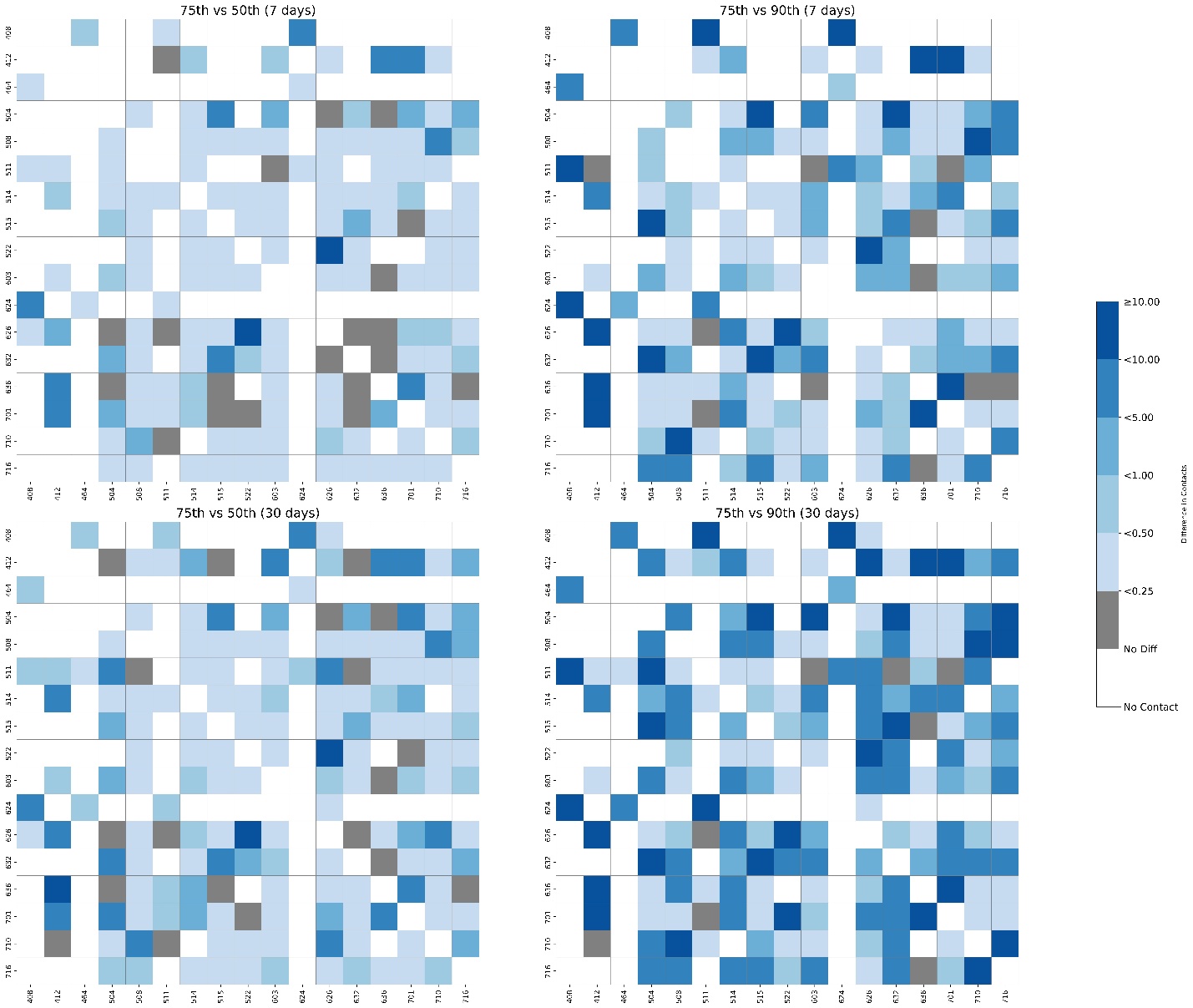


Figure S6 – Contact kernel differences with sensitivity analysis of high-use area definition, with 50^th^ ,75^th^ and 90^th^ percentile.

**Hidden Markov Model for Behavioral State**

In order to infer host behavioral states from GPS movement tracks, we fitted individual three-state Gaussian Hidden Markov Models (HMMs) to each of the collared pigs on the basis of standardized spatial and temporal features. Each model took as input x and y coordinates (UTM-projected) and a normalized timestamp variable, which equated to relative times scaled between 0 and 1. All of the features were z-transformed to zero mean and unit variance prior to model fitting. For each subject, we independently trained an HMM with three hidden states and a full covariance structure using the GaussianHMM function in the Python package hmmlearn. Training was conducted using the Expectation-Maximization algorithm for a maximum of 200 iterations with a convergence tolerance. Where convergence failed due to too little data, that subject was excluded from behavioral modeling.

After convergence, the most probable series of behavioral states was decoded through the Viterbi algorithm. The emergent hidden states were a posteriori annotated as 'resting', 'foraging', and 'moving' based on their movement patterns. Specifically, we computed step length and turning angle distributions for each state and assigned behavioral labels by rank order: the state with the shortest step lengths and highest turning angle variance was labeled as 'resting'; the middle state as 'foraging'; and the state with the longest, most directional steps as 'moving'. This biologically informed partitioning adapts conventions from movement ecology literature (Pohle et al., 2017; Clontz et al., 2021) and aligns with patterns expected in feral pig behavior. Annotated states were then applied to environmentally-mediated contact events by acquisition-relevant or deposition-relevant behavior.


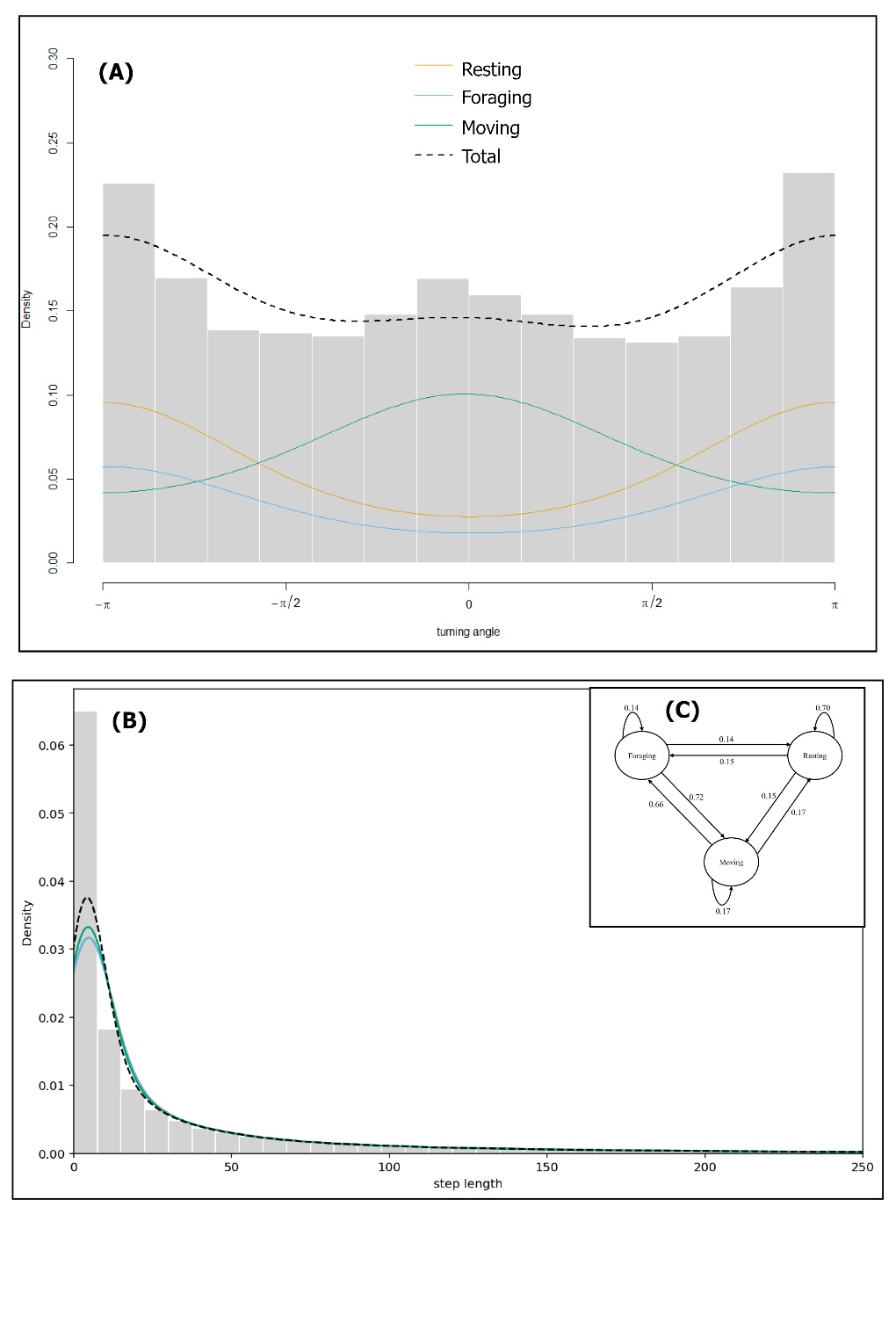


Figure S7: Distribution of the step length and turning angle of the HMM used to determine the behavioral states, (A) Based on turning angle, (B) Based on step length and (C) The transition probability among different states with self-transition.

Pairwise Spatial Distribution of contacts:


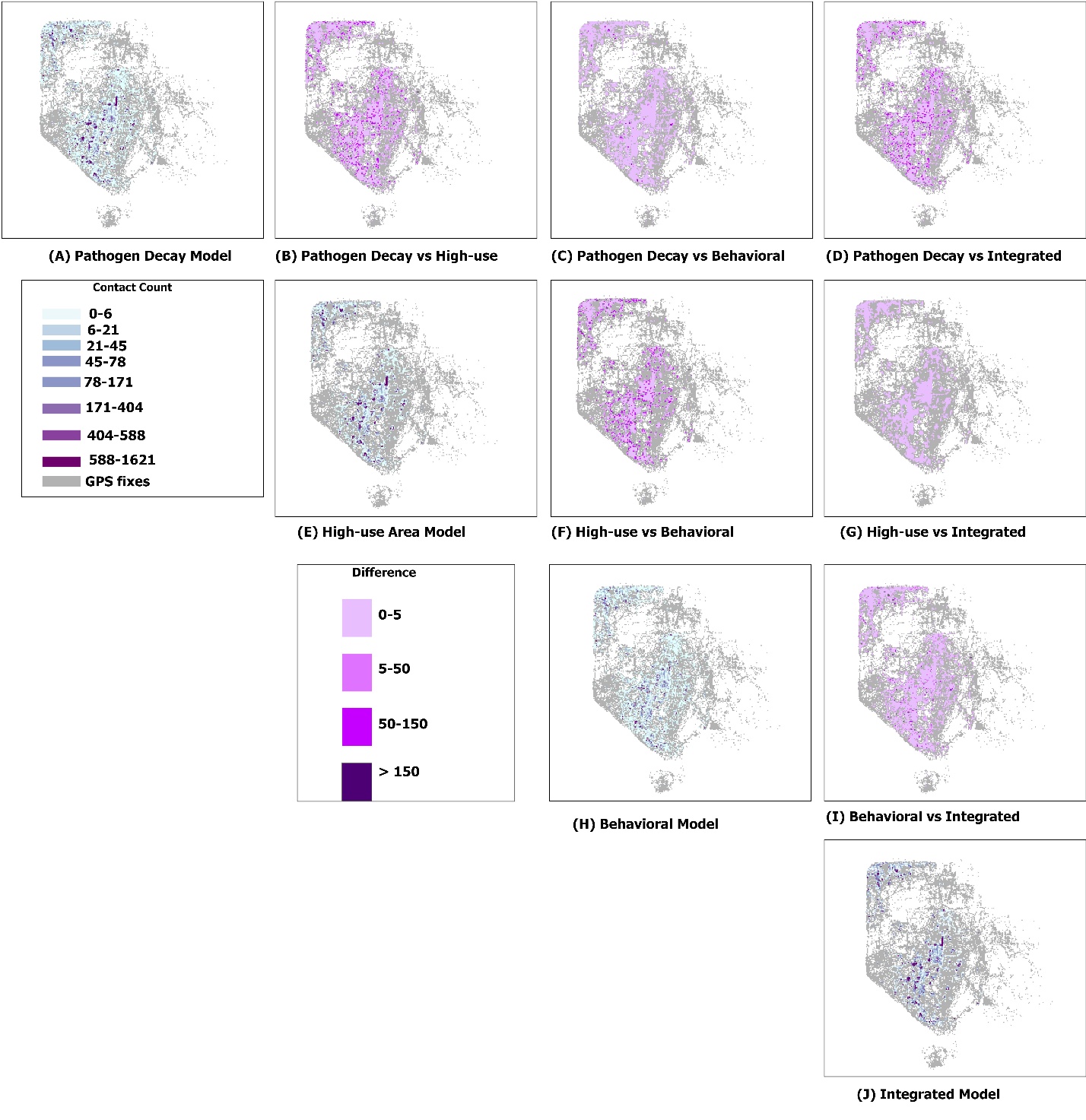


Figure S8: Distribution and difference of contacts among different model for 7-days pathogen decay scenario.


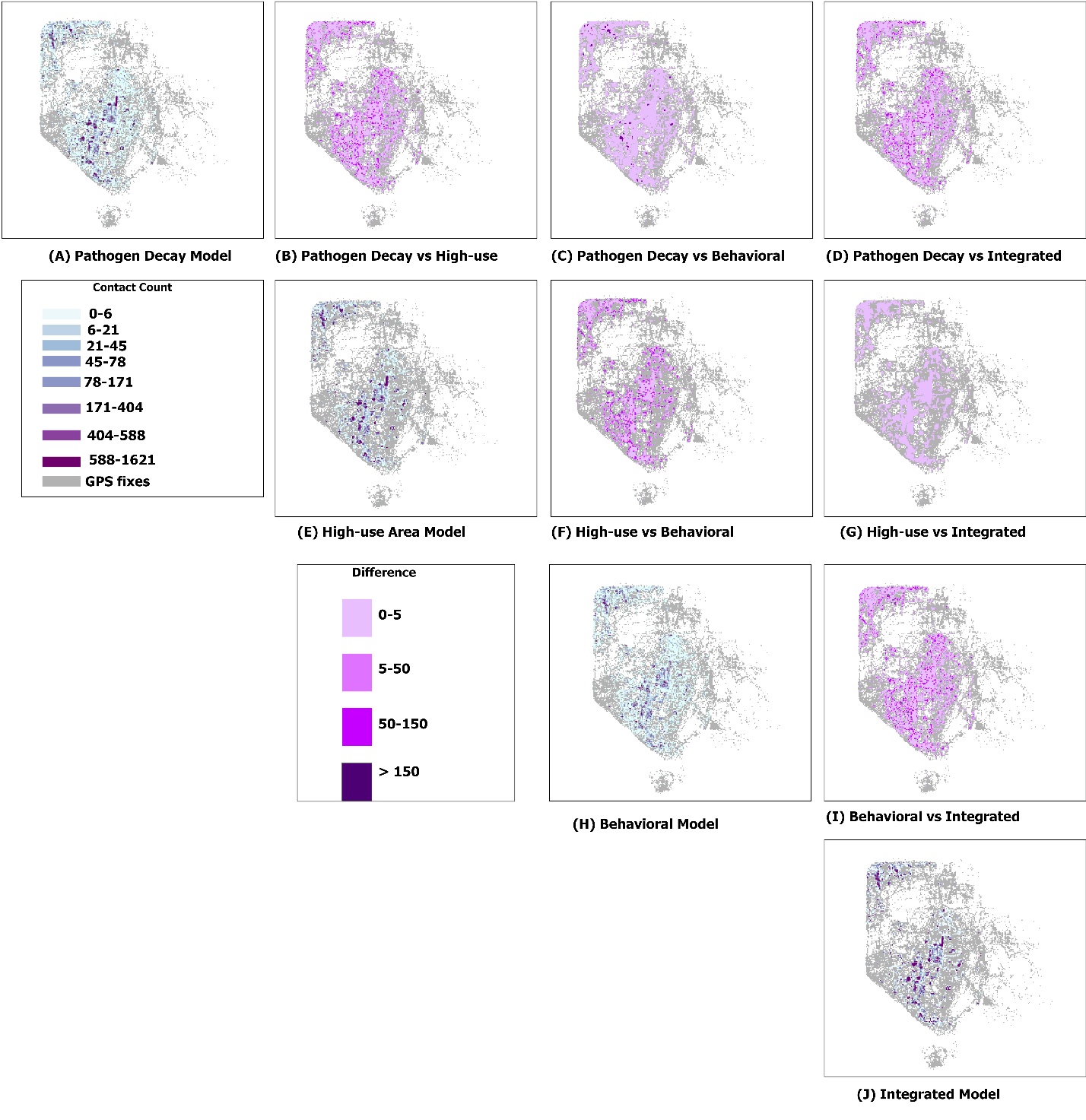


Figure S9: Distribution and difference of contacts among different model for 30-days pathogen decay scenario.

**References:**

Antolin, M. F. 2008. “Unpacking β: Within‐Host Dynamics and the Evolutionary Ecology of Pathogen Transmission.” *Annual Review of Ecology, Evolution, and Systematics* 39, no. 1: 415–437. [**https://doi.org/10.1146/annurev.ecolsys.37.091305.110119**](https://doi.org/10.1146/annurev.ecolsys.37.091305.110119).

Barrios‐Garcia, M. N., and S. A. Ballari. 2012. “Impact of Wild Boar (*Sus scrofa*) in Its Introduced and Native Range: A Review.” *Biological Invasions* 14: 2283–2300. <https://doi.org/10.1007/s10530-012-0229-6>

Bhattarai, A., Villanueva, J., Palekar, R. S., Fagan, R., Sessions, W., Winter, J., & Pennsylvania Working Group. (2011). Viral shedding duration of pandemic influenza A H1N1 virus during an elementary school outbreak—Pennsylvania, May–June 2009. *Clinical Infectious Diseases*, 52(suppl_1), S102–S108. <https://doi.org/10.1093/cid/ciq026>

Brouwer, A. F., Eisenberg, M. C., Shulman, L. M., Famulare, M., Koopman, J. S., Kroiss, S. J., ... & Eisenberg, J. N. (2022). The role of time-varying viral shedding in modelling environmental surveillance for public health: revisiting the 2013 poliovirus outbreak in Israel. *Journal of the Royal Society Interface*, 19(190), 20220006. <https://doi.org/10.1098/rsif.2022.0006>

Buch, D. A., Johndrow, J. E., & Dunson, D. B. (2023). Explaining transmission rate variations and forecasting epidemic spread in multiple regions with a semiparametric mixed effects SIR model. *Biometrics*, 79(4), 2987-2997. <https://doi.org/10.1111/biom.13901>

Dureau, J., Kalogeropoulos, K., & Baguelin, M. (2013). Capturing the time-varying drivers of an epidemic using stochastic dynamical systems. *Biostatistics*, 14(3), 541–555. <https://doi.org/10.1093/biostatistics/kxs052>

Elmonir, W., Abdel-Hamid, N. H., Hamdy, M. E., Beleta, E. I., El-Diasty, M., Melzer, F., Wareth, G., & Neubauer, H. (2022). Isolation and molecular confirmation of *Brucella suis* biovar 2 from slaughtered pigs: an unanticipated biovar from domestic pigs in Egypt. *BMC Veterinary Research*, 18(1), 224. <https://doi.org/10.1186/s12917-022-03332-2>

Miguel, E., Grosbois, V., Caron, A., Pople, D., Roche, B., & Donnelly, C. A. (2020). A systemic approach to assess the potential and risks of wildlife culling for infectious disease control. *Communications Biology*, 3(1), 353. <https://doi.org/10.1038/s42003-020-1032-z>

Podgórski, T., Apollonio, M., & Keuling, O. (2018). Contact rates in wild boar populations: Implications for disease transmission. *The Journal of Wildlife Management*, 82(6), 1210–1218. <https://doi.org/10.1038/nrmicro.2017.45>

Rebollada-Merino, A., Pérez-Sancho, M., Rodríguez-Bertos, A., García, N., Martínez, I., Navarro, A., & García-Seco, T. (2022). Environment and offspring surveillance in porcine brucellosis. *Frontiers in Veterinary Science*, 9, 915692. <https://doi.org/10.3389/fvets.2022.915692>

Silk, M. J., Drewe, J. A., Delahay, R. J., Weber, N., Steward, L. C., Wilson-Aggarwal, J., ... & McDonald, R. A. (2018). Quantifying direct and indirect contacts for the potential transmission of infection between species using a multilayer contact network. *Behaviour*, 155(7–9), 731–757. <https://doi.org/10.1163/1568539X-00003493>

Virlogeux, V., Li, M., Tsang, T. K., Feng, L., Fang, V. J., Jiang, H., & Cowling, B. J. (2015). Estimating the distribution of the incubation periods of human avian influenza A (H7N9) virus infections. *American Journal of Epidemiology*, 182(8), 723–729. <https://doi.org/10.1093/aje/kwv115>
